# TISSUE: uncertainty-calibrated prediction of single-cell spatial transcriptomics improves downstream analyses

**DOI:** 10.1101/2023.04.25.538326

**Published:** 2023-04-29

**Authors:** Eric D. Sun, Rong Ma, James Zou

**Affiliations:** 1 Department of Biomedical Data Science, Stanford University; 2 Department of Statistics, Stanford University

## Abstract

Spatial transcriptomics capture high-resolution spatial distributions of RNA transcripts within biological systems, yet whole-transcriptome profiling of genes at single-cell resolution remains a challenge. To address this limitation, spatial gene expression prediction methods have been developed to infer the spatial expression of unmeasured transcripts, but the quality of these predictions can vary greatly across different contexts. Poor predictions of gene expression in even a small subset of cells or genes can manifest in misleading downstream analyses. As such, there is a need for uncertainty-aware procedures for utilizing predicted spatial gene expression profiles. Here we present TISSUE (Transcript Imputation with Spatial Single-cell Uncertainty Estimation) as a general framework for estimating uncertainty for spatial gene expression predictions and providing uncertainty-aware methods for downstream inference. Leveraging conformal techniques, TISSUE provides well-calibrated prediction intervals for predicted expression values. Moreover it improves downstream analyses to consistently reduce false discovery rates for differential gene expression analysis, improve clustering and visualization of predicted spatial transcriptomics, and improve the performance of predictive models trained on imputed gene expression profiles. We have made TISSUE publicly available as a flexible wrapper method for existing spatial gene expression prediction methods to assist researchers with implementing uncertainty-aware analyses of spatial transcriptomics data.

## 1 Introduction

Spatial transcriptomics technologies extend high-throughput characterization of gene expression to the spatial dimension and have been used to characterize the spatial distribution of cell types and transcripts across multiple tissues and organisms [31, 7, 32, 18, 34, 46]. A major trade-off across all spatial transcriptomics technologies is between the number of genes profiled and the spatial resolution such that most spatial transcriptomics technologies with single-cell resolution are limited to the measurement of a few hundred genes but typically not the whole transcriptome [24]. Given the resource-intensive nature of single-cell spatial transcriptomics data acquisition, computational methods for upscaling the number of genes and/or predicting the expression of additional genes of interest are highly desirable.

There exist several methods for imputing or predicting spatial gene expression using a paired single-cell RNA-seq dataset. Generally, these methods proceed by joint embedding of the spatial and RNA-seq datasets and then predicting expression of new spatial genes by aggregating the nearest neighboring cells in the RNA-seq data [1, 39, 2, 47] or by joint probabilistic modeling, mapping, or transport [27, 8, 44, 46, 11, 33]. For example, SpaGE relies on joint embedding of spatial and RNAseq data using PRECISE domain adaptation followed by k-nearest neighbors regression [35, 1]; a method referred to as “Harmony” here relies on Harmony integration of the two data modalities and averaging of nearest cell expression profiles [2]; and Tangram uses an optimal transport framework with deep learning to devise a mapping between single-cell and spatial transcriptomics data [8]. Applications of these methods have been used in the characterization of spatial differences in aging of mouse neural and glial cell populations [2], recovery of immune signatures in primary tumor samples [44], and identification of spatial patterns in gene regulation [8].

Since the relative performance of these models varies significantly depending on the application area and underlying datasets, there is no best model across all use cases [24]. Moreover, variability in model performance may adversely affect downstream analysis, particularly in promoting false discoveries due to prediction errors. At the same time, few existing gene expression prediction methods provide uncertainty measures for the predicted expression profiles and there are no approaches for utilizing uncertainty in downstream analyses like differential gene expression analysis, hypothesis testing, clustering, visualization, or predictive modeling. As a result, it is often difficult to rely on predicted spatial gene expression profiles without significant external validation or understanding of their context-specific uncertainties.

Here, we present TISSUE (Transcript Imputation with Spatial Single-cell Uncertainty Estimation) as a general wrapper framework around any spatial gene expression imputation or prediction model that produces well-calibrated uncertainty measures that are tailored to the context of each individual model and its use case. We show that TISSUE can be leveraged for improvements in various uncertainty-aware data analysis tasks including the calculation of prediction intervals, hypothesis testing using a multiple imputation approach, clustering and visualization using uncertainty-weighted principal component analysis, and predictive modeling (e.g. cell type classification, anatomic region classification) by uncertainty-aware filtering of cells before training and prediction.

## 2 Results

### 2.1 TISSUE: Cell-centric variability and calibration scores for prediction errors

**Figure 1:**
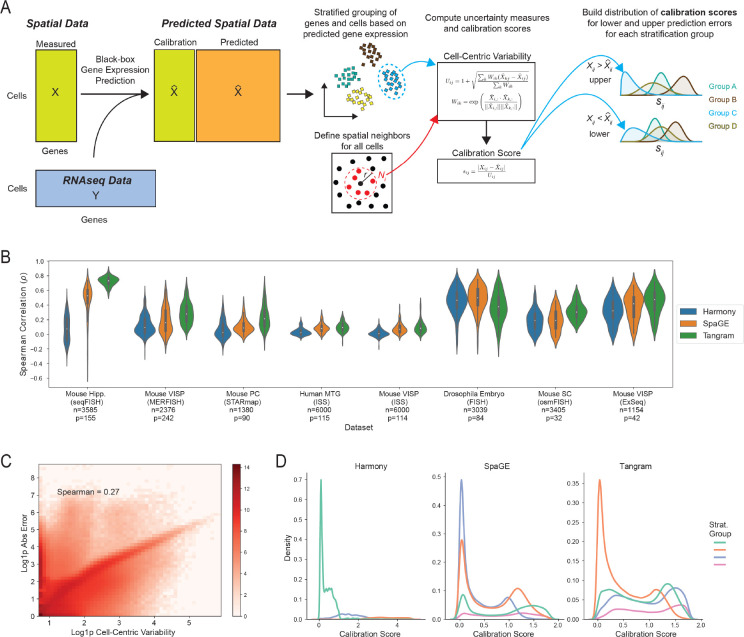
Cell-centric variability and calibration scores for conformal inference. (A) Schematic illustration of the TISSUE calibration score generation pipeline with black-box gene prediction (shown is an example method using paired spatial and RNAseq datasets), stratified grouping of genes and cells, calculation of cell-centric variability measure, and computation and allocation of the calibration score to different stratification groups. (B) Performance of three popular gene prediction methods (Harmony, SpaGE, Tangram) on eight benchmark datasets as measured by the Spearman correlation between predicted and actual gene expression over 10-fold cross-validation. Also shown are the number of cells (n) and number of genes (p) in the spatial transcriptomics datasets. (C) Correlation of TISSUE cell-centric variability and absolute prediction error across all dataset and prediction method combinations computed over 10-fold cross-validation. Log density with added pseudocount (Log1p) is shown by color. (D) Distribution of TISSUE calibration scores on mouse hippocampus ISS dataset and all three prediction method combinations $\left(\left(k_{g},k_{c}\right)=(4,1)\right)$. Details of each dataset and prediction method can be found in Methods.

Spatial gene expression prediction generally relies on leveraging spatial transcriptomics and RNAseq data from similar cell types. The RNAseq data is used to impute the expression of genes not measured in the limited spatial transcriptomics panel and can recover up to whole-transcriptome coverage of genes (Fig. 1A). To motivate the need for uncertainty quantification, we benchmarked three popular spatial gene expression prediction methods (Harmony [2], SpaGE [1], and Tangram [8]) on eight publicly available spatial transcriptomics datasets (spanning seqFISH [29], MERFISH [12], STARmap [45], ISS [21], FISH [23], osmFISH [13], and ExSeq [4] technologies; spatial data visualized in Fig. S1A) paired with single-cell or single-nuclei RNAseq datasets (spanning Smart-seq, Drop-seq, and 10X Chromium technologies) from the same organism and tissue regions [24, 26, 15, 19, 9, 45, 16, 20, 36, 13, 4, 17, 48, 42]. No method consistently outperformed other methods across all spatial transcriptomics datasets. Similarly, methods that performed the best under one metric (e.g. Spearman rank correlation between measured and predicted gene expression, see Fig. 1B) did not necessarily perform the best under an orthogonal evaluation metric (e.g. mean absolute error in predicted expression, see Fig. S1B). For a given method, there is also substantial heterogeneity in the relative performance of the model between genes and cells (Fig. 1A, Fig. S1BC), suggesting that accurate estimation of uncertainty in spatial gene expression predictions may improve confidence in interpretations and downstream analyses.

Here we leverage conformal inference as a natural framework for estimating well-calibrated uncertainties. First, we establish an initial measure of prediction uncertainty that is scalable to unseen observations and agnostic to the prediction error. In order to calibrate these uncertainties to the prediction error, we build distributions of calibration scores by linking these initial measures of uncertainty to the observed prediction errors on existing genes in the spatial transcriptomics data. Finally, these calibration score distributions are used for computing well-calibrated prediction intervals and improving downstream spatial transcriptomics data analysis.

To construct an initial measure of uncertainty that can be universally applied to all existing spatial gene expression prediction methods, we posit that, on average, spatially proximate cells with similar measured gene expression profiles will also have similar expression of genes that are not measured in the spatial transcriptomics gene panel. As a result, large differences in predicted gene expression between neighboring cells of the same cell type would indicate low predictive performance and highly similar predicted gene expressions between neighboring cells would signify high predictive performance for the spatial gene expression prediction method. To quantify this intuition, we introduce the cell-centric variability measure, $U_{ij}$, which, given a gene, computes for each cell a weighted measure of deviation between the predicted expression of that cell and the cells within a spatial neighborhood of it (Eq. (1) and Eq. (2)).


(1) $$U_{ij}=1+\sqrt{\frac{\sum_{k\in N_{i}}W_{ik}{\left({\overset{ˆ}{X}}_{kj}-{\overset{ˆ}{X}}_{ij}\right)}^{2}}{\sum_{k\in N_{i}}W_{ik}}}$$

(2) $$\begin{array}{c} W_{ik}=\exp\left(\frac{{\overset{ˆ}{X}}_{i,:}\cdot {\overset{ˆ}{X}}_{k,:}}{∥{\overset{ˆ}{X}}_{i,:}\left|∥|{\overset{ˆ}{X}}_{k,:}∥\right.}\right) \end{array}$$

Here, ${\overset{ˆ}{X}}_{ij}$ is the predicted gene expression of cell i and gene j. For a given cell i, its spatial neighborhood $N_{i}$ corresponds to the K closest cells in the spatial transcriptomics data according to Euclidean distance. For each neighboring cell k, we compute a weight $W_{ik}$ equal to the exponential cosine similarity in predicted gene expression profiles between the central cell i and its neighbor. These weights prioritize variability in predicted gene expression for similar cells (e.g. cells of the same cell type) and downplays expected variability in gene expression from dissimilar cells without the need to explicitly define cell types or states.

The cell-centric variability is generally positively correlated with the absolute prediction error for spatial gene expression (Fig. 1C). However, the cell-centric variability does not provide an exact estimate of the magnitude of these errors and the relationship between these two quantities is highly context-dependent (Fig. S2A). To explicitly link the cell-centric variability to the prediction error, we adapt a conformal inference framework by computing the calibration score, which is defined as the ratio between the absolute prediction error and the cell-centric variability (Eq. (3), Fig. 1A):

(3) $$s_{ij}=\frac{\left|X_{ij}-{\overset{ˆ}{X}}_{ij}\right|}{U_{ij}},$$

where $X_{ij}$ denotes the measured gene expression for cell i and gene j.

The distribution of $s_{ij}$ can subsequently be used to calibrate uncertainties for new expression predictions by multiplying the cell-centric variability of those predictions by specific quantiles of the $s_{ij}$ calibration score set, which returns values on the scale of prediction errors (see next section for details). To permit flexible calibration schemes within the same spatial transcriptomics dataset, TISSUE allocates calibration scores to disjoint groups of genes and cells, referred to as the stratified calibration sets or groups, which are determined by k-means clustering of genes and then k-means clustering of cells by predicted gene expression (Fig. 1A, see Methods for further details). The number of gene and cell subsets $\left(k_{g},k_{c}\right)$ are user-specified, but downstream results are generally robust to exact specifications of these stratified groupings. Empirically, the distribution of calibration scores can vary significantly across different identified subsets, suggesting the identification of heterogeneous calibration score sets (Fig. 1D and Fig. S2B with $\left(\left(k_{g},k_{c}\right)=(4,1)\right)$. Due to the asymmetric distribution of transcript counts, the calibration scores are further separated by the sign of the prediction error into a lower set for $X_{ij}-{\overset{ˆ}{X}}_{ij}<0$ and upper set for $X_{ij}-{\overset{ˆ}{X}}_{ij}>0$ (Fig. 1A).

### 2.2 TISSUE provides prediction intervals for predicted gene expression

**Figure 2:**
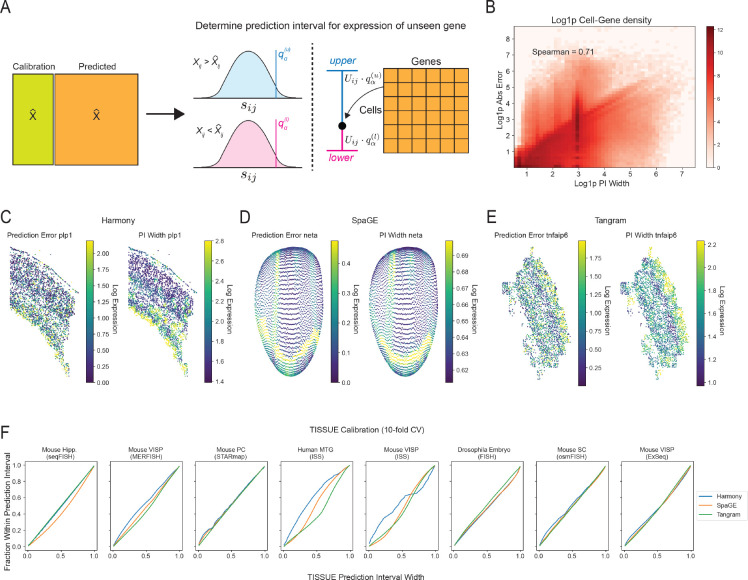
Prediction intervals for spatial gene expression. (A) Schematic illustration of the TISSUE prediction interval retrieval process from the calibration scores for a given confidence level. (B) Correlation of the 67% prediction interval width and the absolute prediction error across all dataset and prediction method combinations computed over 10-fold cross-validation. Log density with added pseudocount (Log1p) is shown by color. (C) Comparison of absolute prediction error (left) and the 67% prediction interval width (right) for a representative gene in the mouse somatosensory cortex osmFISH dataset; (D) in the virtual Drosophila embryo spatial transcriptomics dataset; (E) and in the mouse primary visual cortex MERFISH dataset. (F) Calibration curves for TISSUE prediction intervals showing empirical coverage as a function of the specified confidence level across 10-fold cross-validation. All prediction intervals were generated with $\left(k_{g},k_{c}\right)=(4,\;1)$ settings for stratified grouping.

We leverage a conformal inference framework to convert cell-centric variability of new spatial gene expression predictions into well-calibrated prediction intervals using the calibration scores derived from the measured gene panel. Given a gene expression prediction for cell a and gene b and confidence level α, we compute the cell-centric variability $U_{ab}$ and multiply it by the (⌈(n+1)(1-α)⌉/n)-th quantile of the upper and lower calibration sets of $s_{ij}$ corresponding to all predicted expression values from the same stratified group as the predicted expression of cell a and gene b, which yields the asymmetric TISSUE prediction interval with approximate 1-α coverage (Fig. 2A). Using this approach, TISSUE prediction intervals can be obtained for every predicted gene expression and every cell in the spatial transcriptomics data (see Methods for details and mathematical guarantees). The TISSUE prediction interval width is positively correlated with the absolute prediction error of measured genes under cross-validation (Fig. 2B). For individual genes of interest, the TISSUE prediction interval width generally reflects the spatial distributions of absolute prediction errors such as for *Plp1* in osmFISH profiling of mouse somatosensory cortex (Fig. 2C), *Neta* in a virtual spatial transcriptomics profile of *Drosophila* embryo (Fig. 2D), and *Tnfaip6* in MERFISH profiling of mouse primary visual cortex (Fig. 2E). Averaged across all genes and cells, the TISSUE prediction interval provides well-calibrated coverage of prediction errors on unseen genes for a broad range of confidence levels and across all prediction methods and spatial trancriptomics datasets (Fig. 2F). For individual genes, there is a general tendency towards well-calibrated prediction intervals (Fig. S3A). Similar calibration quality for prediction intervals was also observed under other choices of $k_{g}$ and $k_{c}$ (Fig. S3BC).

### 2.3 Uncertainty-aware hypothesis testing and differential gene expression analysis with TISSUE

**Figure 3:**
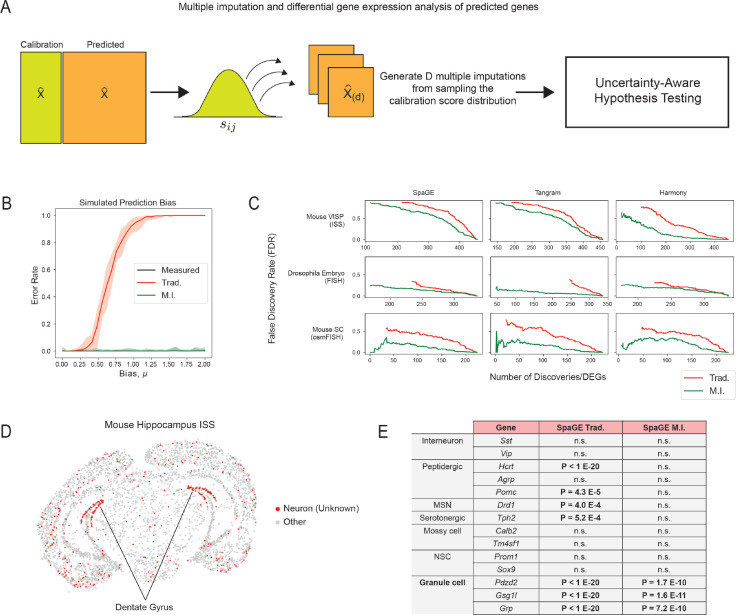
Uncertainty-aware differential gene expression analysis with TISSUE. (A) Schematic illustration of the TISSUE multiple imputation pipeline for hypothesis testing. Calibration scores are randomly sampled and used to compute new predicted gene expression profiles and statistics are compiled across all imputations using a modified two-sample t-test. (B) Error rate of significance testing of gene expression between a homogeneous group of cells (n=1000,p=100) as a function of the bias in prediction error for half of the genes in half of the cells using a Benjamini-Hochberg correction for 5% false discovery rate. Shown are error rates for two-sample t-test on the measured gene expression profiles (black), two-sample t-test on predicted gene expression (Trad., red), and modified two-sample t-test using the multiple imputation approach on predicted gene expression (M.I., green). Results were obtained using stratified grouping settings of $\left(k_{g},k_{c}\right)=(2,2)$. (C) False discovery rate of differentially expressed genes between cell type or anatomic region labels (one versus all approach) using the differentially expressed genes on the measured gene expression profiles as the ground truth across different p-value cutoffs. Shown are results for all three prediction methods and all spatial transcriptomics datasets with cell type or region labels available. All calibration scores were generated with $\left(k_{g},k_{c}\right)=(4,1)$ settings for stratified grouping. (D) Mouse primary visual cortex ISS dataset with the unknown neuronal cluster colored in red and spatially localized to the dentate gyrus of the hippocampus. (E) Marker genes for the unknown neuronal cell cluster are differentially expressed for multiple neuronal cell type gene sets using traditional hypothesis testing with two-sample t-test on the predicted gene expression (SpaGE Trad.), but are selectively differentially expressed for granule cell marker genes using the modified two-sample t-test with multiple imputation from TISSUE calibration scores (SpaGE M.I.). P-values are shown for all predicted neuronal marker genes with significance threshold of Bonferroni-adjusted p<0.05. All calibration scores were generated with $\left(k_{g},k_{c}\right)=(4,1)$ settings for stratified grouping.

Hypothesis testing of differences in gene expression between experimental conditions, cell types, or other groupings is an important tool in understanding biological heterogeneity and perturbation effects using spatial transcriptomics. We extend TISSUE calibration scores for more robust hypothesis testing of differential predicted gene expression across conditions. Specifically, TISSUE hypothesis testing involves sampling multiple imputations for the predicted gene expression values by first sampling calibration scores ${\overset{ˆ}{s}}_{ij}$’s from corresponding calibration sets and then perturbing the original predicted expression values by $U_{ij}\cdot {\overset{ˆ}{s}}_{ij}$ with the direction of the perturbation dependent on whether the sampled score was in the upper or lower set (Fig. 3A). Repeating this process D times yields D possible imputations for each cell and gene. Using multiple imputation theory, TISSUE then derives corrected measures of statistical significance using a modified independent two-sample t-test (Fig. 3A, see Methods for further details). These corrected statistics account for the uncertainty in prediction as encoded by the sampling of scores for generating new imputations. This multiple imputation framework can easily be extended to other statistics of interest [37, 3, 25].

To compare TISSUE hypothesis testing to traditional hypothesis testing using only the predicted gene expression values, we generated synthetic data in which there are two groups of cells with the same ground truth gene expression. The measured gene expression is generated by adding standard Gaussian noise to the ground truth gene expression. Then, predicted gene expression is generated by adding biased Gaussian noise with mean μ to a portion of the genes in one of the cell groups but not the other, and standard Gaussian noise to all other measured gene expression values. Under this context, TISSUE hypothesis testing exhibited near zero error rate in calling differentially expressed genes between the two cell groups $\left(\left(k_{g},k_{c}\right)=(2,2)\right.$, Benjamini-Hochberg corrected p-value cutoff for FDR=5%) across different levels of prediction bias while the error rate quickly saturated under traditional hypothesis testing even for low values of prediction bias (Fig. 3B).

In order to evaluate TISSUE hypothesis testing on real-world data, we compared it to the traditional hypothesis testing approach on three spatial transcriptomics datasets with associated cell type or anatomic region labels (see Methods for details on data labeling). For each label, we computed statistical significance of gene expression differences within that label as compared to all cells with different labels (i.e. one-versus-all approach). Statistical significance was assessed for all genes in the measured gene expression with traditional hypothesis testing and in the predicted gene expression with both TISSUE and traditional hypothesis testing. Using the differentially expressed genes detected using measured gene expression values as the ground truth, we observed lower false discovery rate of differentially expressed genes using TISSUE hypothesis testing as compared to traditional hypothesis testing across all prediction methods and datasets. The lower FDR was observed across for different numbers of differentially expressed gene detections (Fig. 3C) and for different p-value cutoffs (Fig. S4A). This decrease in FDR was also observed across different choices of $k_{g}$ and $k_{c}$ (Fig. S4BC). As such, application of TISSUE hypothesis robustly guards against false discoveries when performing differential gene expression analysis with predicted spatial gene expression profiles.

To illustrate a specific use case of TISSUE hypothesis testing, we applied the method to a ISS mouse primary visual cortex dataset. Using unbiased Leiden clustering, we identified several broad neuronal cell type clusters along with specific non-neuronal cell type clusters. We were unable to further resolve the neuronal cell clusters and used spatial gene prediction with SpaGE to predict the expression of additional neuronal subtype markers that were not in the original ISS gene panel. For one neuronal cluster, which localized to the dentate gyrus of the hippocampus (Fig. 3D) differential expression was detected across most neuronal subtype markers, but under TISSUE hypothesis testing, we observed that this cluster had selective differential expression of predicted marker genes associated with granule cells (*Pdzd2, Gsg1l, Grp*), which are concentrated in the dentate gyrus (DG) of the hippocampus [6], and no significant expression of other cell type markers, including those for mossy cells (*Calb2, Tm4sf1*) and neural stem cells (*Prom1, Sox9*), which are other cell types found in the DG [6], and those for other neuronal subtypes (*Sst, Vip, Hcrt, Agrp, Pomc, Drd1, Tph2*) (Fig. 3E). The identification of this neuronal cell cluster as a granule cell cluster was confirmed by spatial localization of these cells to the hippocampal DG and by further confirmation with measured gene marker *Lrtm4*, which has been previously implicated with granule cell processes [40]. Under traditional differential expression testing with the predicted spatial gene expression values, we were unable to recover the same specificity for granule cell markers. We observed similar but reduced trends for Tangram prediction and lack of significance for any markers under Harmony prediction, likely due to the low performance of that model on this dataset (Fig. 1B).

### 2.4 Uncertainty-aware clustering and separation of labels with TISSUE

**Figure 4:**
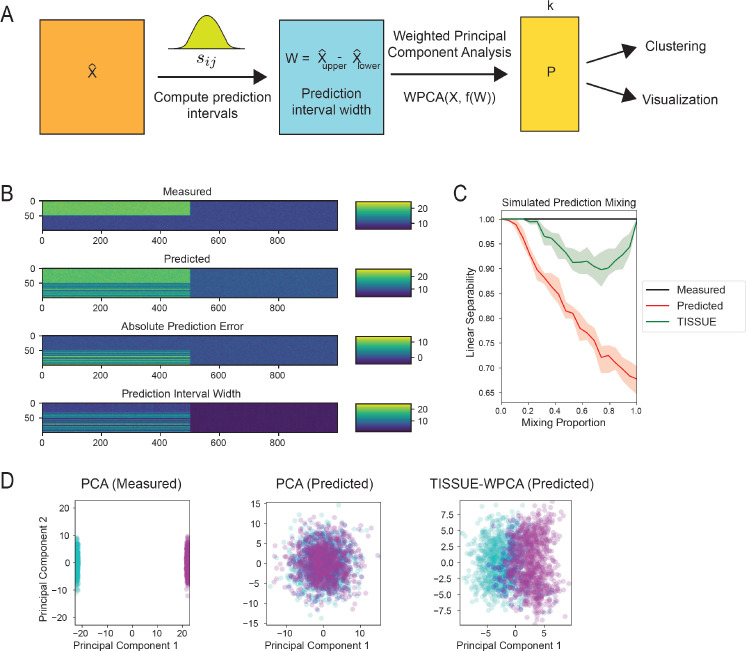
Uncertainty-aware clustering and label separation with TISSUE. (A) Schematic illustration of the weighted principal component analysis (WPCA) pipeline where the inverse TISSUE prediction interval width is used to obtain principal components from WPCA, which are then used for downstream tasks of clustering and label separation. (B) Gene expression profiles for the simulated data visualized by magnitude of expression with rows corresponding to cells and columns corresponding to genes. Shown are the measured gene expression profile simulated by Gaussian noise on top of the true gene expression signal from two distinct cell groups, the predicted gene expression profile with additional Gaussian noise and biased noise added to simulated mixing-in of the top cell cluster into the bottom cell cluster, and the absolute prediction error and 67% prediction interval width obtained through TISSUE. (C) Linear separability measured as the binary classification accuracy of a linear kernel support vector classifier on the two cell clusters as a function of the simulated mix-in proportion. The classifier was trained on the top 15 principal components obtained from the measured gene expression profiles with PCA, predicted gene expression profiles with PCA, and predicted gene expression profiles with TISSUE-WPCA. Results were obtained using stratified grouping settings of $\left(k_{g},k_{c}\right)=(2,2)$. (D) DynamicViz scatter plots of all cells by the first and second principal components from the measured gene expression profiles with PCA, predicted gene expression profiles with PCA, and predicted gene expression profiles with TISSUE-WPCA with complete mixing of the two synthetic cell clusters. DynamicViz visualization was generated by aligning the PCA embeddings from 20 synthetic datasets. Cells are colored to represent the two simulated clusters. Results were obtained using stratified grouping settings of $\left(k_{g},k_{c}\right)=(2,\;2)$.

Cell clustering and visualization are commonly used to identify cell types in spatial transcriptomics data and intuitively understand high-dimensional differences between different metadata labels. TISSUE prediction intervals can be used to generate improved clustering and visualization of predicted gene expression profiles. To build an uncertainty-aware framework for these tasks, we leveraged weighted principal component analysis (WPCA) [14] with weights corresponding to a transformation of the inverse TISSUE prediction interval width for each cell and gene expression prediction (Fig. 4A, see Methods for further details). After applying this TISSUE-WPCA approach to the predicted gene expression data, we then extracted a subset of the top principal components for subsequent clustering and visualization of cells based on their gene expression.

To compare the performance of TISSUE-WPCA to traditional approaches, we generated synthetic spatial transcriptomics data with two distinct cell type clusters where half of the profiled genes are higher in expression in one cell type. Measured data is simulated by adding standard Gaussian noise to the ground truth expression (Fig. 4A). Then, predicted gene expression is simulated by selectively adding biased Gaussian error to a random proportion of cells in one cell type such that the expression profiles of those cells resemble the ground truth of the other cell type (i.e. mix-in bias, Fig. 4A). Using TISSUE, we computed 67% prediction interval widths (analogous to the asymmetric standard error), which were selectively higher in cells with mix-in bias in predicted gene expression and correlated with the absolute prediction error (Fig. 4B). We measured the separability of the two cell type clusters after applying either TISSUE-WPCA or normal PCA to the predicted spatial gene expression profiles by fitting a support vector classifier with a linear kernel to the top 15 principal components and reporting the classification accuracy. TISSUE-WPCA robustly separated the two cell type clusters for all mixing proportions, including up to complete mix-in of the two cell type clusters, while normal PCA observed comparatively lower linear separability for all mixing proportions (Fig. 4C). Using stacked visualization of 20 independent synthetic datasets with DynamicViz [41], we observed that TISSUE-WPCA was able to separate the two cell groups along the first two principal components while the normal PCA was unable to do so under complete mix-in of the two cell groups (Fig. 4D). The DynamicViz variance score was also lower for the TISSUE-WPCA visualization than for the normal PCA visualization (median variance score of 0.223 compared to 0.252), indicating more stable visualization quality in the former, likely due to the improved representation of differences between the two cell type clusters.

Typically, Leiden clustering is used to cluster single-cell and spatial transcriptomics data, often using a subset of the top principal components as input [43]. On all real-world spatial transcriptomics datasets, we observed modest but significant improvement in Leiden clustering quality under TISSUE-WPCA as compared to normal PCA as measured by the adjusted rand index (ARI) with the same Leiden clustering applied to PCA of the measured gene expression serving as the reference clustering (P=0.0164 under paired two-sample t-test for positive mean improvement in ARI using TISSUE-WPCA). The TISSUE-WPCA clustering also reduced the effect of over-clustering that was present for normal PCA on the predicted spatial gene expression profiles with a median number of 17.0 clusters compared to 19.5 clusters respectively where the median number of clusters on the measured gene expression values was 11.0.

### 2.5 Uncertainty-aware predictive modeling with TISSUE

**Figure 5:**
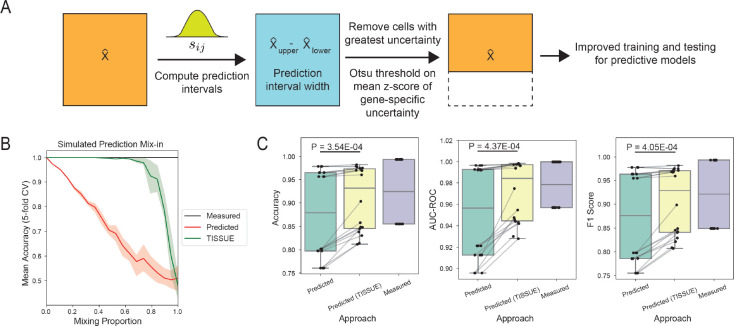
Uncertainty-aware predictive modeling with TISSUE. (A) Schematic illustration of the uncertainty-aware predictive modeling pipeline where the TISSUE prediction interval width is used to filter out cells with poor gene expression predictions from the dataset before training predictive models. (B) Mean accuracy for logistic regression models trained to classify the two cell clusters from the simulated dataset as a function of the mix-in bias. Accuracy was measured across 5-fold cross-validation for models trained and evaluated on the measured gene expression values (black), predicted gene expression values (red), and TISSUE-filtered predicted gene expression values (green). Results were obtained using stratified grouping settings of $\left(k_{g},k_{c}\right)=(2,\;2)$. (C) Accuracy, F1 score, and AUROC (area under the receiver-operator characteristic curve) metrics for logistic regression models trained on the predicted gene expression, TISSUE-filtered predicted gene expression, or measured gene expression for classification of the three most prominent class labels for the mouse somatosensory osmFISH dataset. Shown are metrics for all three prediction methods, three different stratified grouping settings $\left(\left(k_{g},k_{c}\right)=(4,\;1),\left(k_{g},k_{c}\right)=(4,\;2),\left(k_{g},k_{c}\right)=(3,\;3)\right)$, and two sets of class labels (cell type and anatomic region). P-value was computed using a paired two-sample t-test.

Predictive modeling is a common practice with single-cell and spatial transcriptomic data and can lead to useful models for predicting quantities of interest such as biological age [10], cell cycle state [38], and perturbational responses [28]. Substantial errors in spatial gene expression prediction may adversely affect the generalization performance of predictive models trained using the predicted gene expression profiles. We illustrate the utility of TISSUE on improving predictive models in a simple binary classification task using logistic regression. We performed stratified ranking and filtering of cells from the training and evaluation data with respect to the magnitude of uncertainty in their respective classes (Fig. 5A, see Methods for details). On the same synthetic data setup as in Fig. 4A, the classifiers trained and evaluated on TISSUE-filtered data generally outperformed the classifiers trained and evaluated on unfiltered spatial transcriptomics data in separating the two cell groups across different levels of mix-in prediction bias and under cross-validation (Fig. 5B).

To assess TISSUE-filtered training and evaluation on predictive modeling of real-world spatial transcriptomics datasets, we designed two simple classification tasks by retaining cells belonging to the three most prevalent classes of either cell type or anatomic region labels in the osmFISH mouse somatosensory cortex dataset. We then compared the cross-validated performance of logistic regression models trained and evaluated on the TISSUE-filtered predicted spatial gene expression to the performance of logistic regression models trained and evaluated on the unfiltered predicted gene expression profiles. Across three different performance metrics (accuracy, area under the receiver-operator characteristic curve, and F1 score), the TISSUE-filtered classifiers consistently outperformed the unfiltered classifiers on both prediction tasks (Fig. 5C). These trends were generally consistent when aggregating performances across additional spatial transcriptomics datasets for which we derived metadata labels for cell type and anatomic region (Fig. S5, see Methods for details on data labeling).

## 3 Discussion

Spatially resolved transcriptomics at single-cell resolution have paved the way to greater understanding of spatial patterns of gene expression and their underlying biological processes, but are currently limited to a relatively small number of detectable genes. Methods for predicting additional spatial gene expression profiles (e.g. from paired single-cell RNA-seq data) have been proposed to address this limitation. However, these methods can exhibit significant heterogeneity in prediction quality for different cells and different genes, and this heterogeneity manifests differently depending on the selected method and application. The lack of confidence measures for these predictions likely limits the adoption of these methods in spatial transcriptomics data analysis. Here, we developed a computational framework, TISSUE, to compute well-calibrated and context-specific measures of uncertainty for predicted spatial gene expression profiles.

In addition to well-calibrated prediction intervals, TISSUE provides general frameworks for leveraging uncertainty in downstream analysis such as differential gene expression testing, clustering and visualization, and predictive modeling. These frameworks are flexible and can be easily adapted into existing spatial transcriptomics data analysis workflows in place of traditional analysis methods that do not account for uncertainty. For example, the differential gene expression analysis testing approach can be adapted to other hypothesis tests (outside of the two-sample t-test showcased here) by modifying the within-imputation statistics measured for each imputation. Likewise, the weighted principal components obtained from TISSUE-WPCA can be used for any downstream algorithms that utilize the reduced dimensionality representation of PCA as input. Finally, the TISSUE-based filtering of training and evaluation data for predictive modeling strictly modifies the data in a model-agnostic manner and therefore can be extended to both training and deployment of any predictive model across both regression and classification tasks.

TISSUE may have limited performance in contexts where spatial gene prediction patterns are not represented in the calibration set and for genes with extremely sparse expression patterns. Due to the assumptions underlying TISSUE, there may also be reduced performance on rare cell types that are not spatially co-localized. Since TISSUE performance is dependent on the size and diversity of the calibration sets, the method will generally scale better to spatial transcriptomics datasets with a large number of cells, a large number of genes, or more uniform representation of cell types. Although, specification of $k_{g}$ and $k_{c}$ to the natural dimensions of the data based on domain knowledge may provide optimal TISSUE performance, this performance is generally robust to most reasonable choices of $k_{g}$ and $k_{c}$.

The main computational burden imposed by TISSUE is the cross-validated prediction of gene expression on the calibration set, which is necessary for building context-specific uncertainties. For k-fold cross-validation with a given prediction method, TISSUE prediction would take k times longer than a single prediction. Alternative approaches for measuring uncertainty such as variance in predicted expression values over multiple predictions would require significantly more computation than cell-centric variablity to generate predictions for context-specific calibration (i.e. k×l times longer than a single prediction for the variance over l predictions). Here we used 10-fold cross-validation in our experiments, but in practice, the number of cross-validation folds can be specified by the user to achieve a desired runtime. The computational burden for computing cell-centric variability, calibration scores, and prediction intervals is comparatively less than that for generating the initial predictions (Fig. S6).

Although TISSUE has thus far been tested in the spatial transcriptomics setting, the underlying assumptions can generalize to other spatial data modalities, such as spatial proteomics. As whole-transcriptome spatial gene expression profiling becomes possible in the future and other spatial -omics technologies mature, we anticipate that TISSUE will find additional use in the prediction and quantification of uncertainty for enhanced spatial data analysis across multiple modalities.

## 5 Methods

### 5.1 Preprocessing of datasets

We followed data preprocessing approaches from prior benchmark comparisons of spatial gene prediction methods [24], which found highest predictive performance for these methods when non-normalized single-cell spatial transcriptomics data was paired with normalized single-cell RNAseq data. The RNAseq data was normalized using the Scanpy function pp.normalize_total() with its default settings followed by log transformation with an added pseudocount. We selected only genes expressed in at least one percent of cells in the RNAseq data.

### 5.2 Prediction of spatial gene expression

#### 5.2.1 General framework for spatial gene expression prediction

The spatial gene prediction problem involves paired data from spatial transcriptomics and RNA-seq that are approximately from the same tissue and organism. We denote the spatial transcriptomics data as $X\in R^{n\times p}$ and the RNA-seq data as $Y\in R^{m\times q}$, where rows are cells and columns are genes. Generally, spatial gene prediction considers the case where q>>p and the genes present in X are a subset of those in Y. A spatial gene prediction method predicts the expression of a gene that is present in Y but not in X for each cell in X using information from both X and Y.

#### 5.2.2 Harmony

Harmony (as referred to in this work) involves joint embedding of the spatial and RNAseq data using the Harmony algorithm [22] followed by k-nearest-neighbor averaging to calculate predicted expression values for each spatial cell based on its nearest neighbors in the RNAseq data. We implemented the Harmony algorithm following the description outlined in previous applications [2]. We used default Harmony settings in the Scanpy external.pp.harmony_integrate() implementation. We averaged across the 10 nearest RNAseq neighbors for each spatial cell using the first min{30,p} Harmony principal components.

#### 5.2.3 SpaGE

SpaGE performs spatial gene prediction using a two-step approach consisting of alignment using the domain adaptation algorithm PRECISE [35] and then performing k-nearest-neighbor regression [1]. We used a local download of the SpaGE algorithm available at https://github.com/tabdelaal/SpaGE with version corresponding to a download date of July 19, 2022. We set the number of principal vectors in SpaGE equal to 20 if p<40 and to min{n,p}/2 rounded to the nearest integer if p≥40 and otherwise used the default settings.

#### 5.2.4 Tangram

Tangram uses a deep learning framework to create a mapping for projecting RNAseq gene expression onto space [8]. We followed preprocessing details for Tangram according to previous benchmarks [24], which consisted of Leiden clustering on the scaled highly variable genes in the spatial data using Scanpy methods with default settings unless otherwise specified: pp.highly_variable_genes(), sc.pp.scale() with max_value=10, tl.pca(), pp.neighbors (), and tl.leiden () with resolution=0.5. After preprocessing, the identified clusters were used by Tangram to project the RNAseq cells onto space using map_cells_to_space() with mode=’clusters’ and density_prior=’rna_count_based’ and project_genes() with default settings.

### 5.3 Calibration scores for spatial gene expression prediction

#### 5.3.1 Cross-validated spatial gene expression prediction

In order to compute calibration scores, we obtain estimated gene expression predictions on genes that are already measured in the spatial transcriptomics data. This is achieved through a cross-validation approach where a subset of the genes in the spatial transcriptomics data are left out and the gene prediction method is fitted to the remaining genes to make predictions on the left-out subset of genes. In practice, we use 10-fold cross-validation to obtain predictions for all genes in the spatial transcriptomics data but the TISSUE implementation provides options to customize the cross-validation procedure according to user specifications.

#### 5.3.2 Cell-centric variability

We outline three desiderata to guide the development of a scalar uncertainty measure for spatial gene prediction:

1. To ensure computational scalability, the measure can be calculated on a single set of predicted gene expression values.
2. To accurately measure heterogeneity in prediction performance, the measure provides specific values for any cell and gene.
3. The measure ideally leverages spatial and gene expression similarity information.

We introduce the cell-centric variability to satisfy these desiderata. Specifically, for a cell i and gene j, the cell-centric variability $U_{ij}$ is computed according to Eq. (1) and Eq. (2) using cells in its local neighborhood $N_{i}$. We defined cell neighborhoods as the 15 nearest cells by Euclidean distance for each cell, and removed outliers by subsequently excluding neighbors with distance greater than Q3+1.5×(Q3-Q1), where Q3 and Q1 are the third and first quartiles of neighbor distances across all cell neighborhoods. The intercept term in Eq. (1) is included to ensure well-defined calibration scores and non-zero prediction interval widths for cells with no differences in gene expression across its neighborhood, which can result from the high sparsity of single-cell transcriptomics data. The weights $W_{ik}$ in Eq. (2) are used to impose a soft weighting of the cell-centric variability for similar neighboring cells (i.e. of the same cell type) over dissimilar neighboring cells (i.e. of different cell types) without the need for user specification of discrete cell type clusters. The cell-centric variability can be computed for all cells and genes in both the calibration set (genes in the spatial transcriptomics data) and evaluation and test sets (genes to be predicted that are not in the spatial transcriptomics data).

#### 5.3.3 Calculation of calibration scores from variability measure

To link the cell-centric variability to the prediction error, we compute the calibration score as shown in Eq. (3). We compute calibration scores for all pairs of cells and genes in the calibration set (i.e. present in spatial transcriptomics data) and allocate them to their corresponding stratification groups (see following section for details). Calibration scores are further separated by the sign of $X_{ij}-{\overset{ˆ}{X}}_{ij}$ to construct non-symmetric uncertainty bounds around the predicted expression value with $X_{ij}\geq {\overset{ˆ}{X}}_{ij}$ designating inclusion in the calibration scores set for the upper interval and $X_{ij}\leq {\overset{ˆ}{X}}_{ij}$ designating inclusion in the calibration scores set for the lower interval.

#### 5.3.4 Stratified cell and gene grouping for calibration scores

In addition to context-specific construction of calibration scores, TISSUE can also provide finer groupings of genes and cells, each with their own calibration score sets. These stratified groupings are specified by the number of gene groups $k_{g}$ and the number of cell groups $k_{c}$ for a total for $k_{g}\times k_{c}$ groups. Stratified grouping is performed for genes first through k-means clustering with $k=k_{g}$ of the genes on the first 15 principal components representing the transposed predicted gene expression matrix. Then, within each of the identified gene strata, we perform further k-means clustering with $k=k_{c}$ of the cells on the first 15 principal components representing the predicted gene expression matrix restricted to genes present in that strata.

Since there is no guarantee that all stratified groups will contain genes in the calibration set or that all stratified groups will have adequate number of scores for calibration, for stratified groups with less than 100 scores in either the upper interval or lower interval calibration score sets, we defaulted the calibration score set to the union of all calibration score sets across all stratified groups.

### 5.4 Conformal prediction intervals

#### 5.4.1 Retrieval of prediction intervals from calibration scores

For a given confidence level α, we construct the prediction interval with approximate probability coverage (1-α) by retrieving the $\frac{\left\lceil (n+1)(1-\alpha )\right\rceil }{n}$ quantiles of the upper interval calibration scores and lower interval calibration scores. Referring to these quantiles as $q_{\alpha }^{\left(u\right)}$ and $q_{\alpha }^{\left(l\right)}$ respectively, the non-symmetric conformal prediction interval for the predicted gene expression of cell i and gene j can be computed as:

(4) $$I_{ij}=\left[{\overset{ˆ}{X}}_{ij}-U_{ij}\cdot q_{\alpha }^{\left(l\right)},{\overset{ˆ}{X}}_{ij}+U_{ij}\cdot q_{\alpha }^{\left(u\right)}\right]$$

Since this prediction interval does not explicitly depend on the measured prediction error, it can be calculated for all predicted gene expression values, even if the gene was not originally present in the calibration set.

#### 5.4.2 Coverage guarantee for TISSUE prediction intervals

Under regularity conditions, the conformal inference framework provides consistent symmetric prediction intervals when applied to a scalar uncertainty measures such as cell-centric variability [5]. Building on that result, we show that this consistency is still valid with the non-symmetric prediction intervals that we compute using TISSUE.

##### Proposition 1

Let ${\left\{X_{\left[:,j\right]}\right\}}_{j\in [1,\ldots ,p,test]}$ *be i.i.d. from some distribution, then* $P\left(X_{ij}\in \left[l_{ij},u_{ij}\right]\right)\geq 1-\alpha$ *for any confidence level* 0≤α≤1, *where* $l_{ij}$ *and* $u_{ij}$ *are the quantiles of the lower and upper calibration score sets corresponding to* $X_{ij}$.

Here, ‘test’ refers to the index or set of indices for predicted genes that are not in the measured spatial transcriptomics data. Using the notation $l_{ij}={\overset{ˆ}{X}}_{ij}-U_{ij}\cdot q_{\alpha }^{\left(l\right)}$ and $u_{ij}={\overset{ˆ}{X}}_{ij}+U_{ij}\cdot q_{\alpha }^{\left(u\right)}$, the coverage of the TISSUE prediction interval for some confidence level α can be represented as follows:

(5) $$P\left(X_{ij}\in [l_{ij},u_{ij}]\right)\;=P\left(X_{ij}\in [l_{ij},{\overset{ˆ}{X}}_{ij}]\right)+P\left(X_{ij}\in [{\overset{ˆ}{X}}_{ij},u_{ij}]\right)\;\;=P\left(X_{ij}\in [l_{ij},{\overset{ˆ}{X}}_{ij}]|X_{ij}<{\overset{ˆ}{X}}_{ij}\right)P\left(X_{ij}<{\overset{ˆ}{X}}_{ij}\right)+P\left(X_{ij}\in [{\overset{ˆ}{X}}_{ij},u_{ij}]|X_{ij}>{\overset{ˆ}{X}}_{ij}\right)P\left(X_{ij}>{\overset{ˆ}{X}}_{ij}\right)\;\;\geq \left(1-\alpha \right)\left(P\left(X_{ij}<{\overset{ˆ}{X}}_{ij}\right)+P\left(X_{ij}>{\overset{ˆ}{X}}_{ij}\right)\right)\;\;\geq 1-\alpha ,$$

with the first inequality following from Theorem 1 of [5]. And thus, given that symmetric intervals provide proper coverage [5], then we are also guaranteed proper coverage with the asymmetric prediction intervals produced by TISSUE, which is further evident through the empirical coverage assessment for TISSUE (Fig. 2F). This guarantee extends to the stratified group setting for $k_{g}>1$ and/or $k_{c}>1$ (see Proposition 2 in [5]).

#### 5.4.3 Evaluation of prediction intervals

We evaluate the empirical coverage of the prediction intervals using 10-fold cross-validation where the evaluation set of genes in the spatial transcriptomics data is left out of the calibration set and using the calibration scores, we compute conformal prediction intervals for all cells and genes in the evaluation set. We then compute empirical coverage of the conformal prediction intervals as the fraction of measured gene expression values in the evaluation set that fall within the conformal prediction interval across all cells and genes and alternatively across all cells for each gene with nonzero predicted and measured expression. The empirical coverage is computed separately for the calibration set as a standard of comparison.

### 5.5 Uncertainty-aware hypothesis testing for predicted spatial gene expression

#### 5.5.1 Generating multiple imputations using calibration scores

We introduce a multiple imputation procedure for generating uncertainty-aware hypothesis testing for predicted spatial gene expression. Multiple imputations are generated by uniformly sampling calibration scores from the corresponding union of upper and lower interval calibration score sets for each predicted spatial gene expression value. Given a uniform random sample of such calibration scores, $S_{\left(d\right)}\in R^{n\times p}$, we compute an alternative imputation as:

(6) $${\overset{ˆ}{X}}_{\left(d\right)}=\overset{ˆ}{X}+\delta_{u/l,(d)}*S_{\left(d\right)}*U$$

where * denotes element-wise multiplication, $U\in R^{n\times p}$ is equal to the cell-centric variability measures computed on $\overset{ˆ}{X}$, and $\delta_{u/l}=1$ if the score was sampled from the upper interval set and $\delta_{u/l}=-1$ if the score was sampled from the lower interval set. This sampling is repeated D-1 times to generate D multiple imputations including the original imputation $\overset{ˆ}{X}$. We tempered the multiple imputations against outliers by restricting the sampling to the scores within the set corresponding to the 80% conformal prediction interval.

#### 5.5.2 Modified two-sample t-test for multiple imputation

After generating D multiple imputations from the calibration scores, we perform hypothesis testing using a modified two-sample t-test. Consider two groups with sets of sample/cell indices A and B. Then, the mean difference and variance under normal two-sample t-test for a single imputation are:

(7) $$\mu_{d}=\frac{1}{n_{A}}\sum_{i\in A}{\hat{X}}_{i,:}^{\left(d\right)}-\frac{1}{n_{B}}\sum_{i\in B}{\hat{X}}_{i,:}^{\left(d\right)}$$

(8) $$s_{d}^{2}=\left(\frac{1}{n_{A}}+\frac{1}{n_{B}}\right)\left(\frac{\left(n_{A}-1\right)Var\{{\overset{ˆ}{X}}_{i,:}^{\left(d\right)}:i\in A\}+\left(n_{B}-1\right)Var\{{\overset{ˆ}{X}}_{i,:}^{\left(d\right)}:i\in B\}}{n_{A}+n_{B}-2}\right),$$

where ${\overset{ˆ}{X}}^{\left(d\right)}$ denotes the d-th imputation among the D multiple imputations. Extending these statistical measures to the multiple imputation case, we use the standard modification for multiple imputation [37, 30, 3, 25], which results in the following mean and variance:

(9) $$\mu_{MI}=\frac{1}{D}\sum_{d=1}^{D}\mu_{d}$$

(10) $$s_{MI}^{2}=s_{W}^{2}+\left(1+\frac{1}{D}\right)s_{B}^{2}$$

where $s_{W}^{2}$ is the within-imputation variance and $s_{B}^{2}$ is the between-imputation variance, computed as:

(11) $$s_{W}^{2}=\frac{1}{D}\sum_{d=1}^{D}s_{d}^{2}$$

(12) $$s_{B}^{2}=\frac{1}{D-1}\sum_{d=1}^{D}{\left(\mu_{d}-\mu_{MI}\right)}^{2}.$$

Then, the modified test statistics for the two-sample t-test is:

(13) $${\tilde{t}}_{MI}=\frac{\mu_{MI}}{\sqrt{s_{MI}^{2}}},$$

which is t-distributed with degrees of freedom $(D-1){\left(1+\frac{Ds_{W}^{2}}{(D+1)s_{B}^{2}}\right)}^{2}$ and the resulting probability can be interpreted as the posterior probability of a significant difference in means between the two groups (i.e. β≠0) accounting for both evidence of this effect and the reliability of the imputations by inflating the standard error for this effect [37]. Under some regularity assumptions (approximate normality of imputed values and missing at random), the multiple imputation approach produces consistent estimates [3, 25].

### 5.6 Simulated prediction bias for differential gene expression analysis

We generated synthetic data for comparing the uncertainty-aware hypothesis testing approach against traditional hypothesis testing. First, we created a ground truth spatial gene expression matrix consisting of n=100 cells and p=1000 genes with zero expression values for each instance. The first 50 cells were identified as under the first condition A and the remaining 50 cells were identified as under the second condition B. The spatial locations of the cells spanned a 10 × 10 grid where each cell was placed in ascending index across rows (left to right) and moving upwards (bottom to top). To simulate measurement error, we added standard Gaussian noise (mean equal to zero, variance equal to one) to the ground truth expression. Then, to simulate prediction bias for cells under condition B, we added shifted Gaussian noise (mean equal to μ≥0, variance equal to one) to the measured values of the last 500 genes for all cells in condition B. Standard Gaussian noise was added to the measured values for the other cells. Under this simulation, there are no true differences between the cells in condition A as compared to condition B in the ground truth and virtually no differences detected in the measured gene expression value but differences are observed after prediction due to added bias. TISSUE, which is calibrated on prediction error of the calibration genes, is able to group and estimate more appropriate prediction intervals based on the spatial localization of these biased prediction errors.

### 5.7 Cell type annotation procedure for mouse VISP MERFISH dataset

We preprocessed the data using a standard Scanpy pipeline. Starting with the counts matrix, we normalized the data using pp.normalize_total() with default settings, log-transformed the data using pp.log1p(), and scaled the data with pp.scale(). We computed principal components and a neighbors graph using tl.pca() followed by pp.neighbors() with 20 principal components. Finally, we performed Leiden clustering using tl.leiden() with resolution of 0.1, which yielded 11 cell clusters. We used tl.rank_genes_groups() with the Wilcoxon method to identify the top five marker genes for each cell cluster and manually identified the clusters using those markers. In total, we identified endothelial cells, oligodendrocytes, astrocytes, and eight neuron-like cell clusters.

### 5.8 Anatomic region annotation procedure for Drosophila embryo dataset

We used the same preprocessing procedure as for the mouse VISP MERFISH dataset. We identified seven Leiden clusters and grouped them into four region labels based on their spatial localization with two “posterior” clusters, one “anterior” cluster, one “bottom” cluster, and three “middle” clusters.

### 5.9 Weighted PCA for uncertainty-aware clustering

We implemented a weighted version of principal component analysis where each value in the gene expression matrix is assigned a scalar weight. We compute the weights according to the following steps. First, we compute the inverse of the TISSUE prediction interval width (i.e. 67% prediction interval upper bound minus lower bound). Then, we normalize these values for each gene by the mean value across that gene to correct for expression level differences between genes. Finally, we binarize these normalized values so that the top 80% of normalized values will have ten-fold higher relative weight than the bottom 20% of normalized values. These binary values are used as weights for Weighted Principal Component Analysis (WPCA). WPCA directly decomposes the weighted covariance matrix to obtain principal vectors, and then applies weighted least squares optimization to retrieve the principal components [14]. We used the implementation of WPCA in the wpca (v.0.1) Python package with default settings and weights set according to our specification.

To compute separability or clustering results from the WPCA principal components, we used the first 15 principal components as input either in the Leiden clustering algorithm (using Scanpy’s pp.neighbors() followed by tl.leiden() with default settings) or in fitting a support vector classifier with linear kernel (using Scikit-Learn’s svm.SVC() with kernel=‘linear’). To obtain ground truth labels for the Leiden clustering on the measured gene expression, we normalized and log-transformed the gene expression values and then performed PCA and Leiden clustering using the same specifications as before.

### 5.10 Dynamic visualization of first two principal components

We generated dynamic visualizations of the first two principal components for visual comparison of PCA on the measured spatial gene expression, PCA on the predicted spatial gene expression, and WPCA on the predicted spatial gene expression. We used DynamicViz (v.0.0.3) to center and rigidly align the cells across 20 synthetic datasets, visualized the resulting alignments using viz.stacked(), and scored their variability by computing variance scores for each cell using score.variance() with method=’global’.

### 5.11 Simulated prediction bias for clustering and predictive modeling

We generated synthetic data for benchmarking the uncertainty-aware clustering/separation and predictive modeling approaches. First, we created a ground truth spatial gene expression matrix consisting of n=100 cells and p=1000 genes with zero expression values for each instance. The first 50 cells were identified as cell type A and the remaining 50 cells were identified as cell type B. All cells of cell type A have ground truth expression of 20 for the first 500 genes and expression of 10 for the other 500 genes; cells of cell type B have ground truth expression of 10 for all 1000 genes. The spatial locations of the cells spanned a 10 × 10 grid where each cell was placed in ascending index across rows (left to right) and moving upwards (bottom to top). To simulate measurement error, we added standard Gaussian noise (mean equal to zero, variance equal to one) to the ground truth expression. Then, to simulate prediction bias for cells in cell type B, we added shifted Gaussian noise (mean equal 10, variance equal to one) to the first 500 genes of a proportion of the cells in cell type B sampled randomly and determined by the specified ‘mix-in‘ proportion. For the sampled cells, this simulated prediction bias shifts their predicted gene expression profiles to be more similar to that of cell type A rather than cell type B. Standard Gaussian noise was added to all other measured values for both cell types.

### 5.12 Uncertainty-aware cell filtering for predictive modeling

Using the TISSUE prediction interval, we perform filtering of cells in training and/or evaluation data for predictive modeling. First, we convert all gene expression values to z-scores using the mean and standard deviation of expression for each gene in the data. Then, for each cell, we assign a score based on its average z-score across all genes. The cells with the highest scores are removed from the filtered data. The threshold for removal is automatically determined using Otsu’s method, which finds a threshold that maximizes the variance between the filtered and unfiltered score sets. In the context of classification, we avoid inter-class differences in prediction uncertainty by performing this filtering procedure independently within each class.

## Supplementary Material

Supplement 1

## Data Availability

Raw data were accessed from existing benchmark datasets [24] and are available from the following studies: Mouse Hippocampus: Spatial transcriptomics (seqFISH) at https://content.cruk.cam.ac.uk/jmlab/SpatialMouseAtlas2020/; RNAseq (10X Chromium) at GSE158450 in GEO for ‘HIPP_sc_Rep1_10X sample’. Mouse VISP: Spatial transcriptomics (MERFISH) at https://github.com/spacetx-spacejam/data/; RNAseq (Smart-seq) at https://portal.brain-map.org/atlases-and-data/rnaseq/mouse-v1-and-alm-smart-seq for mouse primary visual cortex (VISp). Mouse Prefrontal Cortex (PC): Spatial transcriptomics (STARmap) at ‘20180419_BZ9_control’ in https://www.starmapresources.com/data; RNAseq (10X Chromium) at GSE158450 in GEO for ‘PFC_sc_Rep2_10X’. Human Middle Temporal Gyrus (MTG): Spatial transcriptomics (ISS) at https://github.com/spacetx-spacejam/data; RNAseq (Smart-seq) at https://portal.brain-map.org/atlases-and-data/rnaseq/human-mtg-smart-seq. Mouse VISP: Spatial transcriptomics (ISS) at https://github.com/spacetx-spacejam/data; RNAseq (Smart-seq) at https://portal.brain-map.org/atlases-and-data/rnaseq/mouse-v1-and-alm-smart-seq for mouse primary visual cortex (VISp). Drosophila Embryo: Spatial transcriptomics (FISH) at https://github.com/rajewsky-lab/distmap; RNAseq (Drop-seq) at GSE95025 in GEO. Mouse Somatosensory Cortex (SC): Spatial transcriptomics (osmFISH) at http://linnarssonlab.org/osmFISH/ for cortical region subset; RNAseq (Smart-seq) at https://portal.brain-map.org/atlases-and-data/rnaseq/mouse-whole-cortex-and-hippocampus-smart-seq for mouse somatosensory cortex (SSp). Mouse VISP: Spatial transcriptomics (ExSeq) at https://github.com/spacetx-spacejam/data; RNAseq (Smart-seq) at https://portal.brain-map.org/atlases-and-data/rnaseq/mouse-v1-and-alm-smart-seq for mouse primary visual cortex (VISp).
