# Supplementary material for: TISSUE: uncertainty-calibrated prediction of single-cell spatial transcriptomics improves downstream analyses
Source: bioRxiv. 2023 Sep 3:2023.04.25.538326. Originally published 2023 Apr 29. Preprint. [Version 2] doi: 10.1101/2023.04.25.538326 (PMC10168375; doi:10.1101/2023.04.25.538326)
Supplement: 1 [file NIHPP2023.04.25.538326V2-supplement-1.pdf]

bioRxiv preprint doi: https://doi.org/10.1101/2023.04.25.538326; this version posted September 3, 2023. The copyright holder for this preprint (which was not certified by peer review) is the author/funder, who has granted bioRxiv a license to display the preprint in perpetuity. It is made available under aCC-BY 4.0 International license.

6 Extended Data Figures

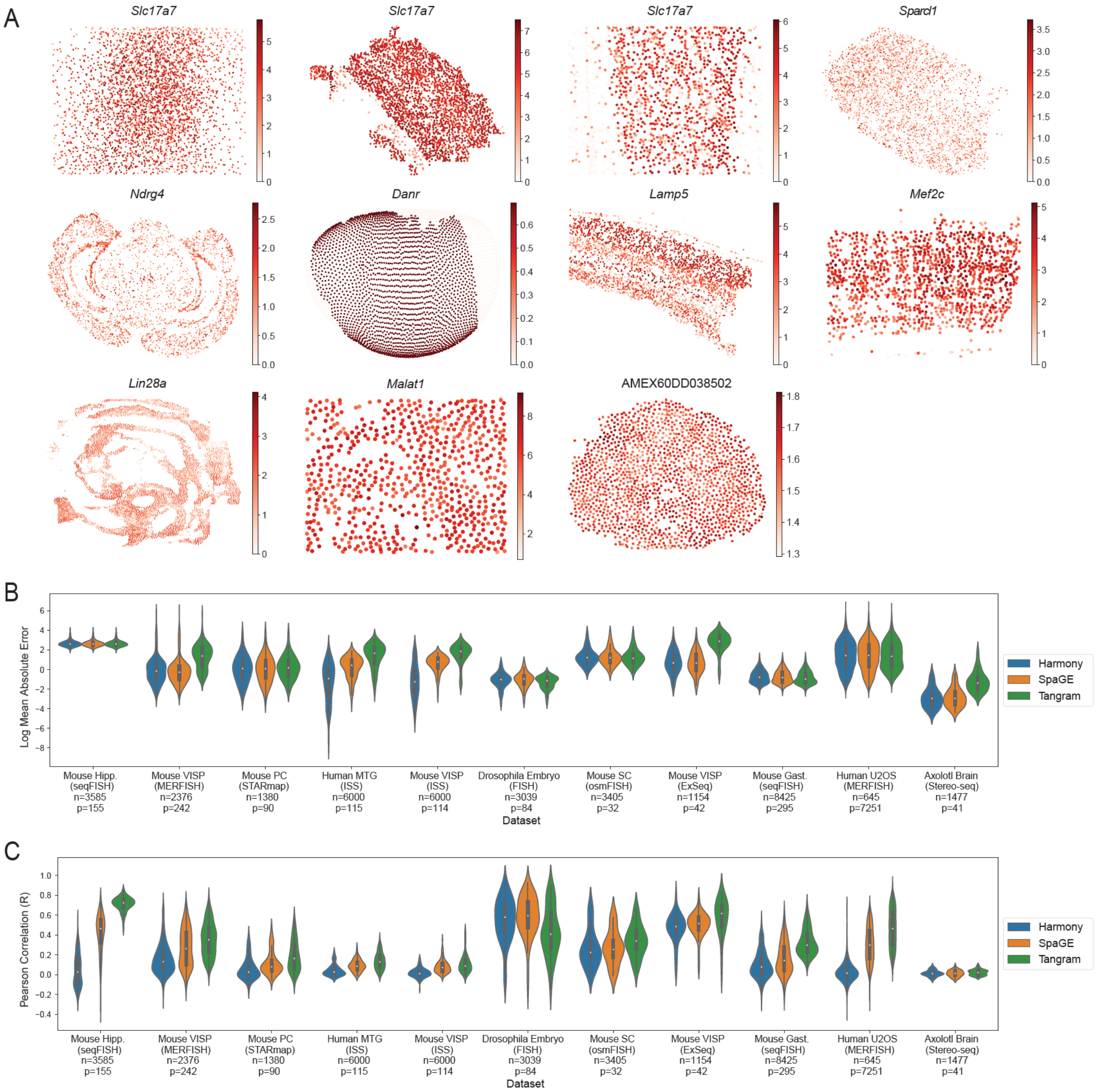

Figure S1: Overview of datasets and prediction performance. (A) Visualization of cells in the eleven spatial transcriptomics datasets colored by the expression of the highest-expressed gene in each respective dataset. (B-C) Performance of all three gene prediction methods (Harmony, SpaGE, Tangram) on all datasets as measured by (B) gene-wise mean absolute error between predicted and actual gene expression over 10-fold cross-validation, and (C) gene-wise Pearson correlation between predicted and actual gene expression over 10-fold cross-validation. Shown also are the number of cells (n) in the spatial transcriptomics datasets and the number of genes (p) shared between spatial and RNAseq datasets. In panels B-C, the inner box corresponds to quartiles of the metrics and the whiskers span up to 1.5 times the interquartile range of the metrics.

bioRxiv preprint doi: https://doi.org/10.1101/2023.04.25.538326; this version posted September 3, 2023. The copyright holder for this preprint (which was not certified by peer review) is the author/funder, who has granted bioRxiv a license to display the preprint in perpetuity. It is made available under aCC-BY 4.0 International license.

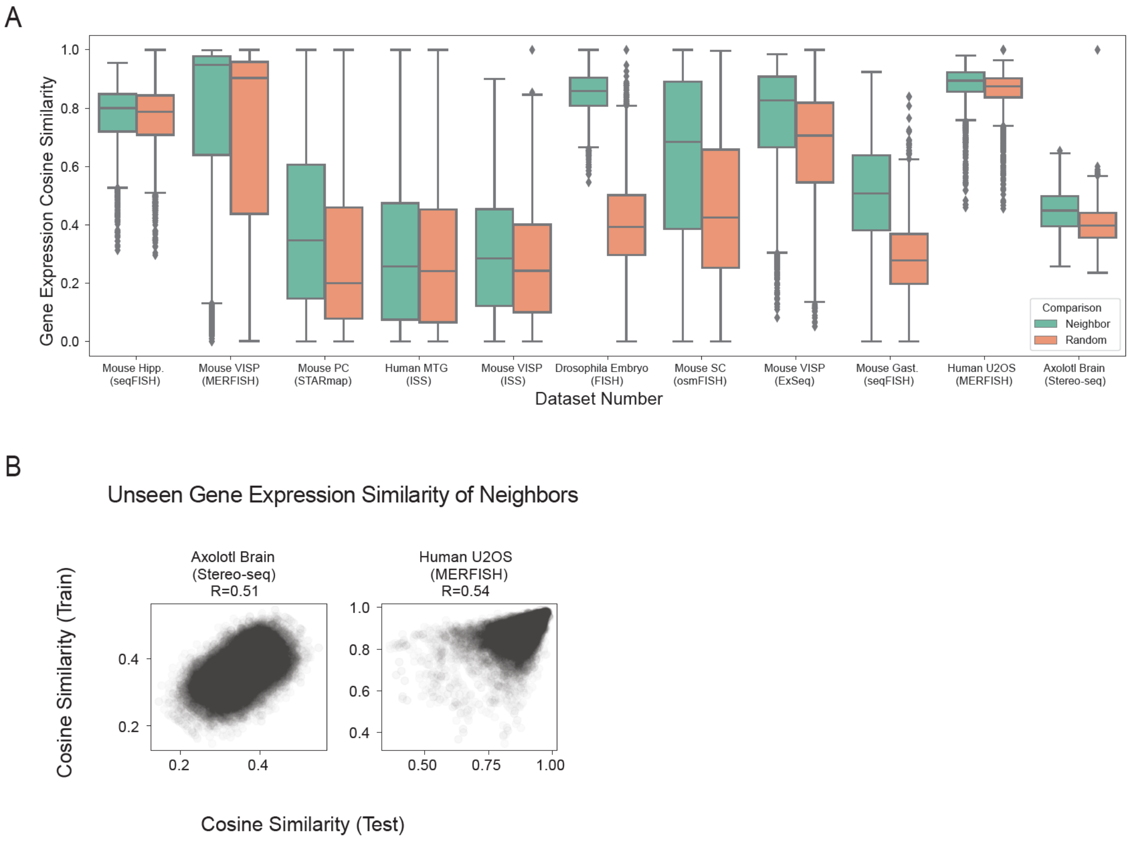

Figure S2: Evidence of gene expression similarity between spatial neighbors. (A) Cosine similarity of gene expression profiles for 250 cells paired with all their neighbors in the TISSUE spatial graph compared to pairings with randomly drawn cells across all eleven spatial transcriptomics datasets. The boxplot corresponds to the quartiles of the cosine similarity measurements. The center line corresponds to median cosine similarity, which was strictly higher in the neighbor-paired comparisons than the random-paired comparisons across all datasets. Whiskers span up to 1.5 times the interquartile range of the metrics and values outside this range are shown as dots. (B) Scatter plots of the cosine similarities of gene expression profiles for 250 cells paired with their neighbors for either the training gene set or the test gene set determined by random train-test split of all genes (50% train, 50% test). Shown are cosine similarity pairs for 10 train-test splits for the two datasets with the most genes.

bioRxiv preprint doi: https://doi.org/10.1101/2023.04.25.538326; this version posted September 3, 2023. The copyright holder for this preprint (which was not certified by peer review) is the author/funder, who has granted bioRxiv a license to display the preprint in perpetuity. It is made available under aCC-BY 4.0 International license.

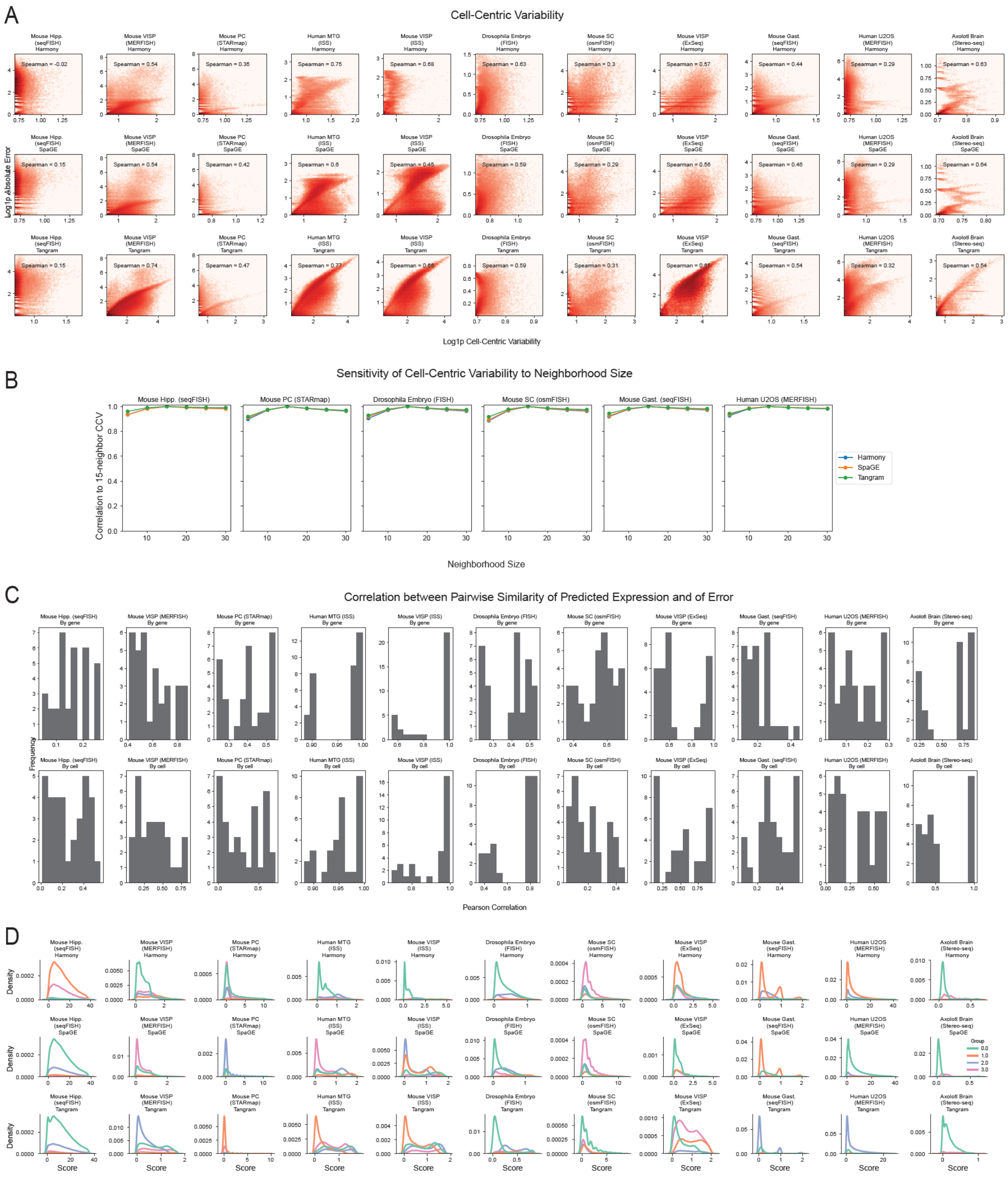

Figure S3: Cell-centric variability and calibration score distributions for individual datasets and prediction methods. (A) Correlation of cell-centric variability and absolute prediction error shown individually for each dataset and prediction method combination computed over 10-fold cross-validation. Log density with added pseudocount (Log1p) is shown by color, with a maximum of 1000 cells and 300 genes sampled from each dataset to provide more uniform representation. (B) Pearson correlation of all cell-centric variability measures obtained for different numbers of neighbors in building the TISSUE spatial graph compared to the default setting of 15 neighbors. Shown are results across six representative datasets. (C) Histograms showing the distribution of Pearson correlations between either gene-wise or cell-wise similarities of prediction errors and similarities of predicted expression values across all spatial transcriptomic datasets. (D) Distribution of TISSUE calibration scores shown individually for each dataset and prediction method combination ($(k_g, k_c) = (4, 1)$). Details on each dataset and prediction method can be found in Methods.

bioRxiv preprint doi: https://doi.org/10.1101/2023.04.25.538326; this version posted September 3, 2023. The copyright holder for this preprint (which was not certified by peer review) is the author/funder, who has granted bioRxiv a license to display the preprint in perpetuity. It is made available under aCC-BY 4.0 International license.

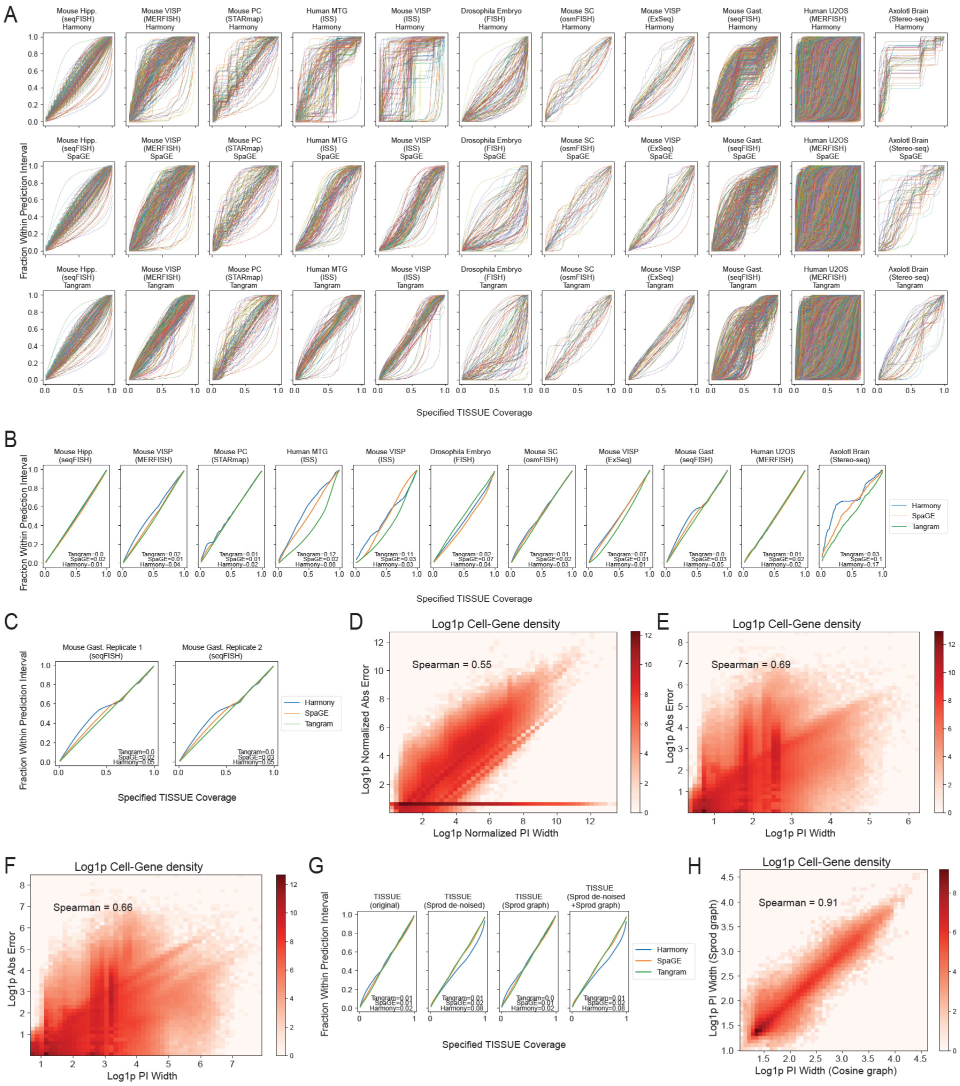

Figure S4: Further evaluation of TISSUE prediction intervals. (A) Gene-level calibration curves for TISSUE prediction intervals showing empirical coverage as a function of the specified confidence level across 10-fold cross-validation. Each line corresponds to an independent gene in the spatial transcriptomics dataset. (B-C) Calibration curves for TISSUE prediction intervals showing empirical coverage as a function of the specified confidence level across 10-fold cross-validation (B) under automated setting of (k_g, k_c) for stratified grouping; and (C) for two technical replicates of the mouse gastrulation seqFISH dataset with $(k_g, k_c) = (4, 1)$. The calibration error is annotated for each prediction method (see Methods). (D-F) Correlation plots across all dataset and prediction method combinations computed over 10-fold cross-validation for (D) the 67% prediction interval width and absolute prediction error, both normalized by the absolute value of the predicted expression; (E) 50% prediction interval width and absolute prediction error; (F) 80% prediction interval width and absolute prediction error. Log density with added pseudocount (Log1p) is shown by color, with a maximum of 1000 cells and 300 genes sampled from each dataset to provide more uniform representation. (G) Calibration curves for TISSUE prediction intervals showing empirical coverage as a function of the specified confidence level across 10-fold cross-validation for the mouse somatosensory cortex osmFISH dataset with different combinations of Sprod de-noising or Sprod-based spatial similarity graph instead of the TISSUE spatial neighbors graph. The calibration error is annotated for each prediction method (see Methods). (H) Correlation plot of 67% prediction interval width with TISSUE spatial neighbors graph with cosine similarity weighting and 67% prediction interval width with Sprod similarity graph and weighting for the mouse somatosensory cortex osmFISH dataset and all prediction methods computed over 10-fold cross-validation.

bioRxiv preprint doi: https://doi.org/10.1101/2023.04.25.538326; this version posted September 3, 2023. The copyright holder for this preprint (which was not certified by peer review) is the author/funder, who has granted bioRxiv a license to display the preprint in perpetuity. It is made available under aCC-BY 4.0 International license.

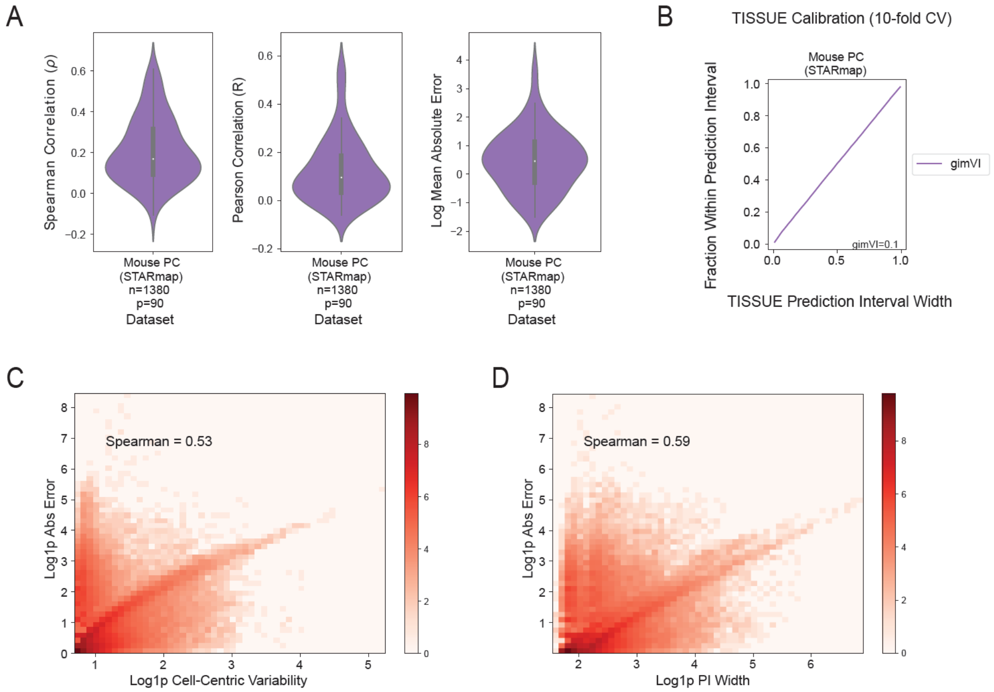

Figure S5: TISSUE results using gimVI for spatial gene expression prediction. (A) Violin plots showing the gene-wise performance of gimVI on predicting the gene expression of the mouse prefrontal cortex STARmap dataset using Spearman correlation (left), Pearson correlation (middle), and mean absolute error (right). The inner box corresponds to the quartiles of the bounds. (B) Calibration curve for TISSUE prediction intervals of gimVI showing empirical coverage as a function of the specified confidence level across 10-fold cross-validation for the mouse prefrontal cortex STARmap dataset with $(k_g, k_c) = (4, 1)$. The calibration error is annotated for each prediction method (see Methods). (C) Correlation plot of cell-centric variability and the mean absolute prediction error of gimVI on the mouse prefrontal cortex STARmap dataset. (D) Correlation plot of 67% prediction interval width and the mean absolute prediction error of gimVI on the mouse prefrontal cortex STARmap dataset. Log density with added pseudocount (Log1p) is shown by color.

bioRxiv preprint doi: https://doi.org/10.1101/2023.04.25.538326; this version posted September 3, 2023. The copyright holder for this preprint (which was not certified by peer review) is the author/funder, who has granted bioRxiv a license to display the preprint in perpetuity. It is made available under aCC-BY 4.0 International license.

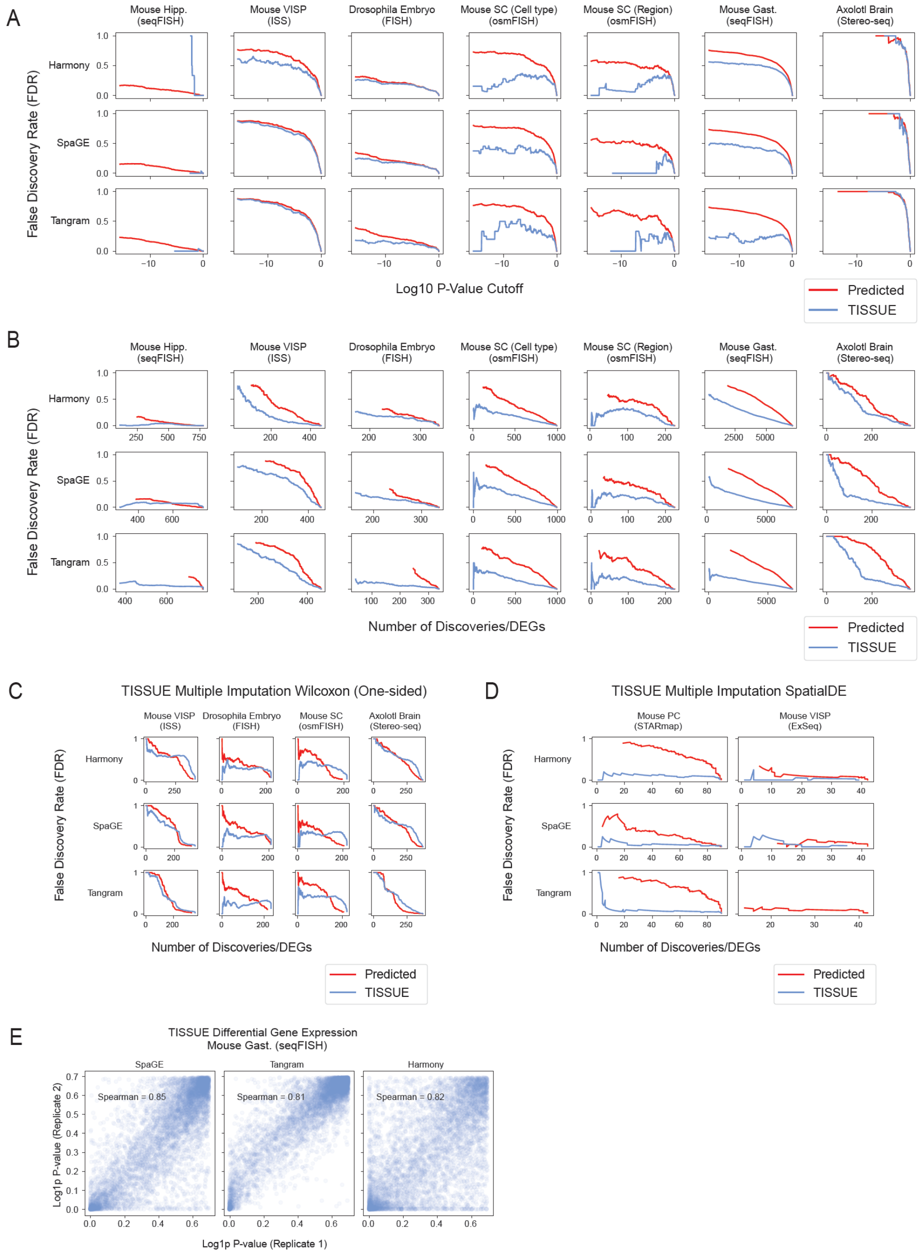

Figure S6: Additional differential gene expression analysis with TISSUE. (A) False discovery rate of differentially expressed genes between cell type or anatomic region labels (one versus all approach) as a function of the p-value threshold for significance and with $(k_g, k_c) = (4, 1)$ settings for stratified grouping.(B) False discovery rate of differentially expressed genes between cell type or anatomic region labels (one versus all approach) as a function of the number of discoveries and with automated stratified grouping. (C) False discovery rate of differentially expressed genes between cell type or anatomic region labels (one versus all approach) as a function of the number of discoveries and with $(k_g, k_c) = (4, 1)$ settings for stratified grouping for the alternative TISSUE multiple imputation framework using the "greater than" one-sided, two-sample Wilcoxon/Mann-Whitney test. (D) False discovery rate of spatially variable genes as a function of the number of discoveries and with $(k_g, k_c) = (4, 1)$ settings for stratified grouping for the alternative TISSUE multiple imputation framework using the SpatialDE test. (E) Correlation plot of the log p-values obtained from the TISSUE multiple imputation t-test framework between two technical replicates of the mouse gastrulation seqFISH dataset.

bioRxiv preprint doi: https://doi.org/10.1101/2023.04.25.538326; this version posted September 3, 2023. The copyright holder for this preprint (which was not certified by peer review) is the author/funder, who has granted bioRxiv a license to display the preprint in perpetuity. It is made available under aCC-BY 4.0 International license.

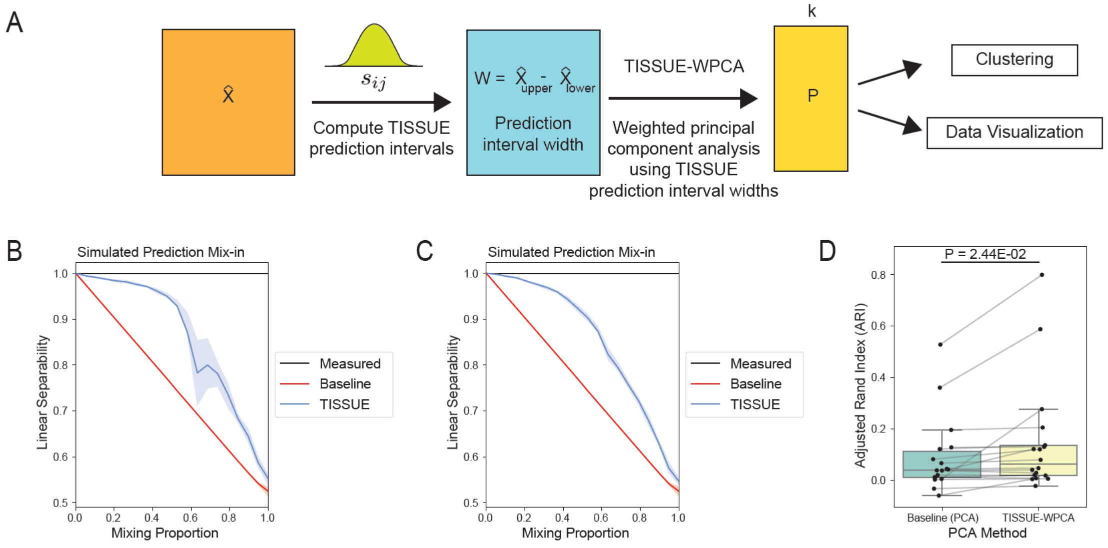

Figure S7: Uncertainty-aware clustering and label separation with TISSUE-WPCA. (A) Schematic illustration of the weighted principal component analysis (WPCA) pipeline where the inverse TISSUE prediction interval width is used to obtain principal components from WPCA, which are then used for downstream tasks of clustering and label separation. (B) Linear separability measured as the binary classification accuracy of a linear kernel support vector classifier fitted on the two cell clusters in the simulated spatial transcriptomics data as a function of the simulated mix-in proportion. The classifier was trained on the top 15 principal components obtained from the measured gene expression profiles with PCA, predicted gene expression profiles with PCA, and predicted gene expression profiles with TISSUE-WPCA. For TISSUE-WPCA, weights were determined by binarizing the inverse normalized 67% prediction interval width (see Methods). Results were obtained using automated stratified grouping. Bands represent the interquartile range and solid line denotes the median linear separability across 20 simulated datasets. (C) Same as in panel B except with TISSUE-WPCA weighting using the log-transformed inverse normalized 67% prediction interval width. (D) Adjusted rand index (ARI) for k-means clustering ($k = 3$) on the top 15 principal components obtained from PCA on the predicted expression or TISSUE-WPCA on the predicted gene expression for six real spatial transcriptomics dataset and labe pairings and all prediction methods. P-value was computed using a paired two-sample t-test. The box corresponds to quartiles of the metrics and the whiskers span up to 1.5 times the interquartile range of the metrics.

bioRxiv preprint doi: https://doi.org/10.1101/2023.04.25.538326; this version posted September 3, 2023. The copyright holder for this preprint (which was not certified by peer review) is the author/funder, who has granted bioRxiv a license to display the preprint in perpetuity. It is made available under aCC-BY 4.0 International license.

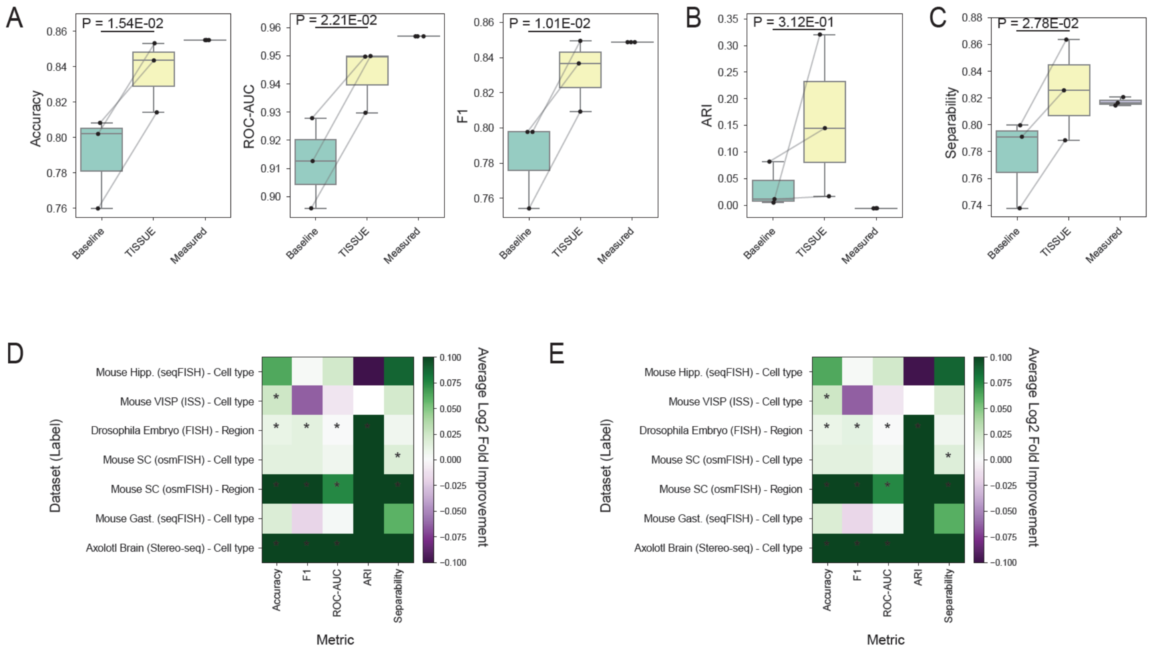

Figure S8: Additional experiments for uncertainty-aware supervised learning, clustering, and visualization. (A-C) Downstream task performance metrics for the three most prominent anatomic region class labels for the mouse somatosensory osmFISH dataset. Shown are metrics for all three prediction methods with automated stratified grouping settings. P-value was computed using a paired two-sample t-test. The box corresponds to quartiles of the metrics and the whiskers span up to 1.5 times the interquartile range of the metrics. (A) Accuracy, F1 score, and ROC-AUC (receiver-operator characteristic area under the curve) metrics for logistic regression models trained on the predicted gene expression, TISSUE-filtered predicted gene expression, or measured gene expression for classification. (B) Adjusted rand index (ARI) for k-means clustering ($k = 3$) on the top 15 principal components obtained from the predicted gene expression, TISSUE-filtered predicted gene expression, or measured gene expression for classification. (C) Linear separability measured as classification accuracy of linear kernel support vector classifier fitted on the top 15 principal components obtained from the predicted gene expression, TISSUE-filtered predicted gene expression, or measured gene expression for classification. (D) Log2 fold change in improvement of performance metrics using TISSUE-filtered PCA in lieu of regular PCA on predicted expression for supervised learning (logistic regression models trained to predict class labels; Accuracy, F1, ROC-AUC), clustering (k-means clustering on top 15 principal components; Adjusted Rand Index (ARI)), and visualization (linear separability measured as the classification accuracy of a linear kernel support vector classifier fitted on the top 15 principal components) for the top three classes across all dataset and class label combinations. Results were obtained using the 50% prediction interval width for filtering. (E) Same as panel E except with the 80% prediction interval width for filtering.

bioRxiv preprint doi: https://doi.org/10.1101/2023.04.25.538326; this version posted September 3, 2023. The copyright holder for this preprint (which was not certified by peer review) is the author/funder, who has granted bioRxiv a license to display the preprint in perpetuity. It is made available under aCC-BY 4.0 International license.

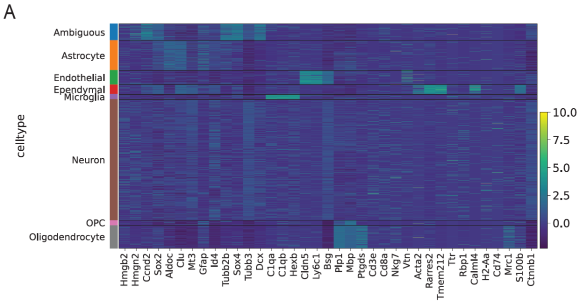

B

	Gene	P-value (Predicted)	P-value (TISSUE)
**Quiescent neural stem cell/astrocyte (qNSC/astrocyte)**	*Slc1a3*	P = 1.4 E-6	n.s.
	Nr2e1	P = 8.4 E-15	n.s.
	Sox9	P = 5.8 E-10	P = 7.6 E-4
	Vcam1	P = 1.4 E-3	n.s.
	Hes5	P = 6.9 E-7	n.s.
	Prom1	P = 3.4 E-4	n.s.
	Thbs4	P = 2.3 E-5	n.s.
	Gja1	P = 1.1 E-5	n.s.
**Activated neural stem cell/neural progenitor cell (aNSC/NPC)**	*Pclaf*	P < 1 E-20	P = 1.2 E-9
	H2afx	P < 1 E-20	P = 9.2 E-8
	Rrm2	P < 1 E-20	P = 2.0 E-7
	Insm1	P = 1.8 E-15	P = 1.2 E-4
	Egfr	P < 1 E-20	n.s.
	Mcm2	P < 1 E-20	n.s.
	Cdk1	P < 1 E-20	P = 4.7 E-6

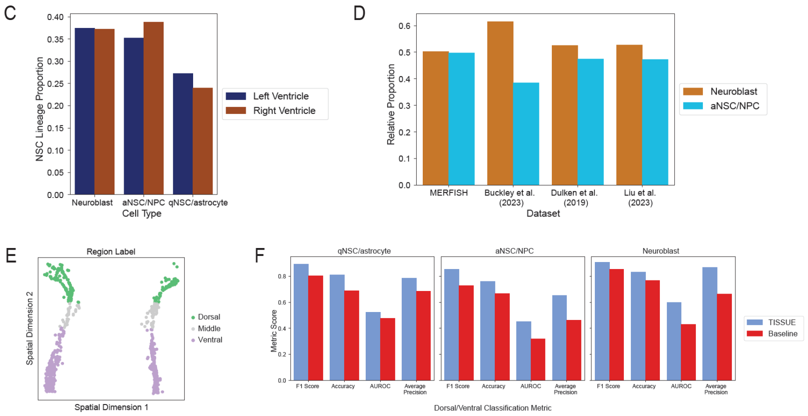

Figure S9: TISSUE is necessary to identify ambiguous NSC lineage subtype. (A) Heatmap of the scaled log-normalized gene expression of original cell type markers in the adult mouse subventricular zone MERFISH dataset for each of the identified cell type clusters. The Ambiguous cell type cluster in the first row exhibits high expression of qNSC/astrocyte, aNSC/NPC, and neuroblast markers. (B) Additional predicted marker genes for the second ambiguous subcluster are differentially expressed for all qNSC/astrocyte and aNSC/NPC markers under traditional hypothesis testing with two-sample t-test on the predicted gene expression (Predicted). With TISSUE multiple imputation t-test, there are substantially more aNSC/NPC markers that are differentially over-expressed in the ambiguous subcluster (TISSUE), permitting identification of this subcluster as an aNSC/NPC subtype cluster. P-values are shown for all predicted marker genes with significance threshold of Bonferroni-adjusted $p < 0.1$. (C) Relative proportion of each of the three TISSUE-identified subtypes in the neural stem cell lineage cluster for either the left or right lateral ventricle. (D) Relative proportions of aNSC/NPC and neuroblast populations across the MERFISH dataset and three single-cell RNAseq datasets of the mouse subventricular zone. The qNSC/astrocyte proportions were not compared since they were aggregated with astrocytes of the striatum in the single-cell RNAseq datasets. (E) Spatial visualization of the cells in the neural stem cell lineage cluster colored by dorsal or ventral spatial location labels. (F) Dorsal versus ventral classification performance of TISSUE-filtered penalized logistic regression models and baseline unfiltered penalized logistic regression models evaluated using 10-fold cross-validation across F1 score, accuracy, area under the receiver-operator curve, and average precision.

bioRxiv preprint doi: https://doi.org/10.1101/2023.04.25.538326; this version posted September 3, 2023. The copyright holder for this preprint (which was not certified by peer review) is the author/funder, who has granted bioRxiv a license to display the preprint in perpetuity. It is made available under aCC-BY 4.0 International license.

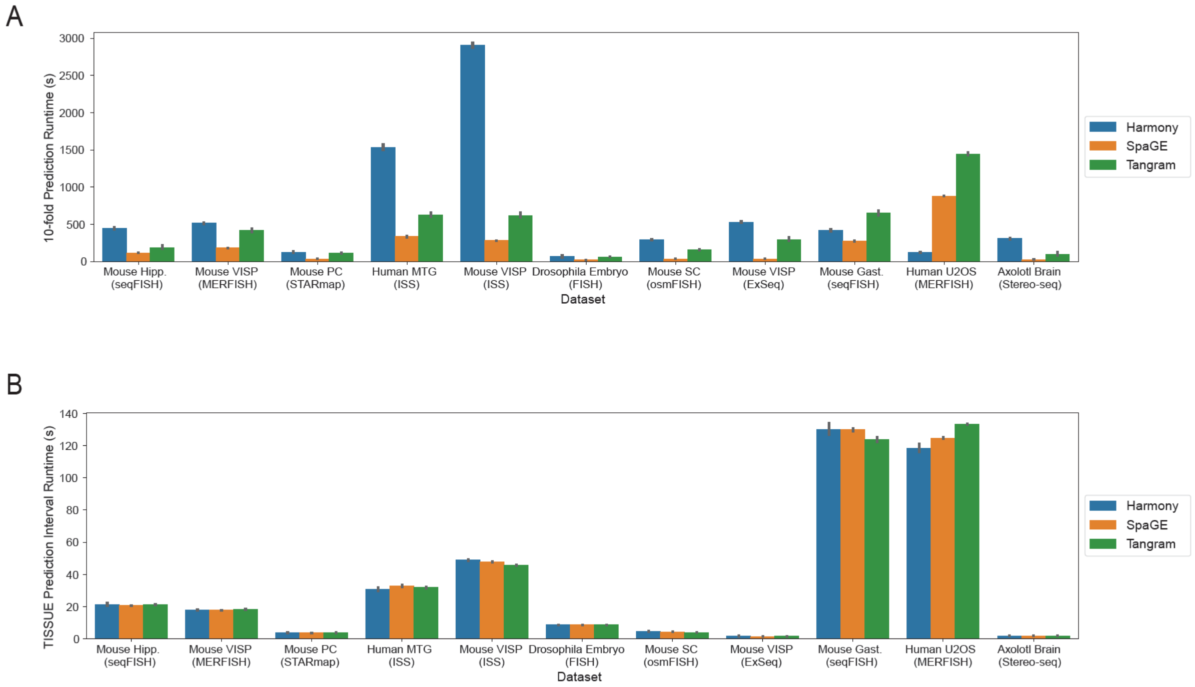

Figure S10: Computational runtime for TISSUE. (A) Bar plots of total runtimes for spatial gene expression prediction computations over 10 predictions to generate estimated predictions on all calibration genes. Error bars correspond to 95% confidence interval over an outer 10-fold cross-validation. (B) Bar plots of total runtimes for TISSUE prediction interval calculation including computation of cell-centric variability and calibration score sets. Error bars correspond to 95% confidence interval over an outer 10-fold cross-validation.
