# Supplementary material for: TISSUE: uncertainty-calibrated prediction of single-cell spatial transcriptomics improves downstream analyses
Source: bioRxiv. 2023 Apr 29:2023.04.25.538326. Preprint. [Version 1] doi: 10.1101/2023.04.25.538326 (PMC10168375; doi:10.1101/2023.04.25.538326)
Supplement: Supplementary file 1 [file NIHPP2023.04.25.538326v1-supplement-1.pdf]

bioRxiv preprint doi: https://doi.org/10.1101/2023.04.25.538326; this version posted April 29, 2023. The copyright holder for this preprint (which was not certified by peer review) is the author/funder, who has granted bioRxiv a license to display the preprint in perpetuity. It is made available under aCC-BY 4.0 International license.

6 Extended Data Figures

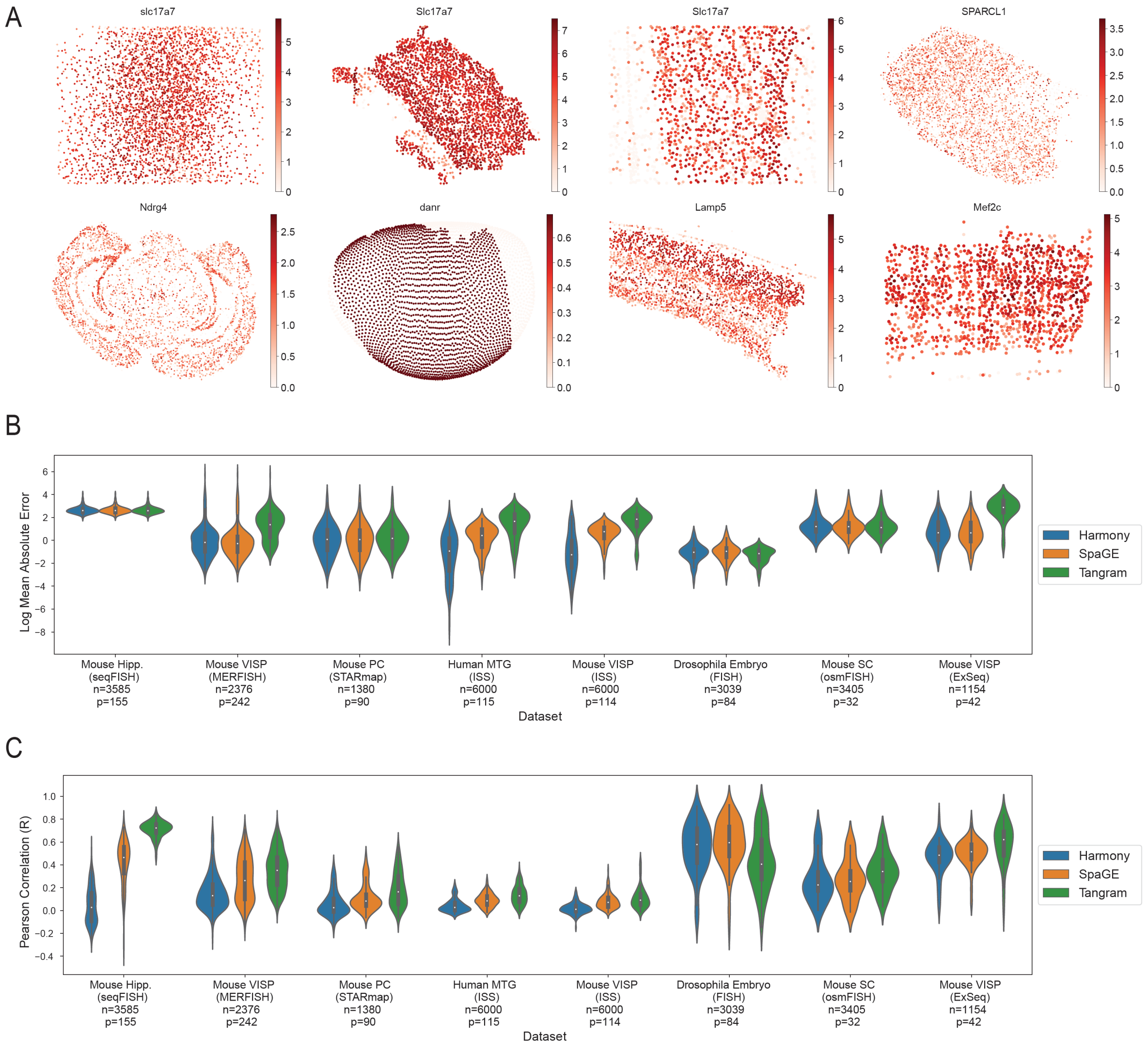

Figure S1: Overview of datasets and prediction performance. (A) Visualization of cells in the eight spatial transcriptomics datasets colored by the expression of the highest-expressed gene in each respective dataset. (B-C) Performance of all three gene prediction methods (Harmony, SpaGE, Tangram) on all eight datasets as measured by: (B) mean absolute error between predicted and actual gene expression over 10-fold cross-validation, and (C) Pearson correlation between predicted and actual gene expression over 10-fold cross-validation. Shown also are the number of cells (n) and number of genes (p) in the spatial transcriptomics datasets.

bioRxiv preprint doi: https://doi.org/10.1101/2023.04.25.538326; this version posted April 29, 2023. The copyright holder for this preprint (which was not certified by peer review) is the author/funder, who has granted bioRxiv a license to display the preprint in perpetuity. It is made available under aCC-BY 4.0 International license.

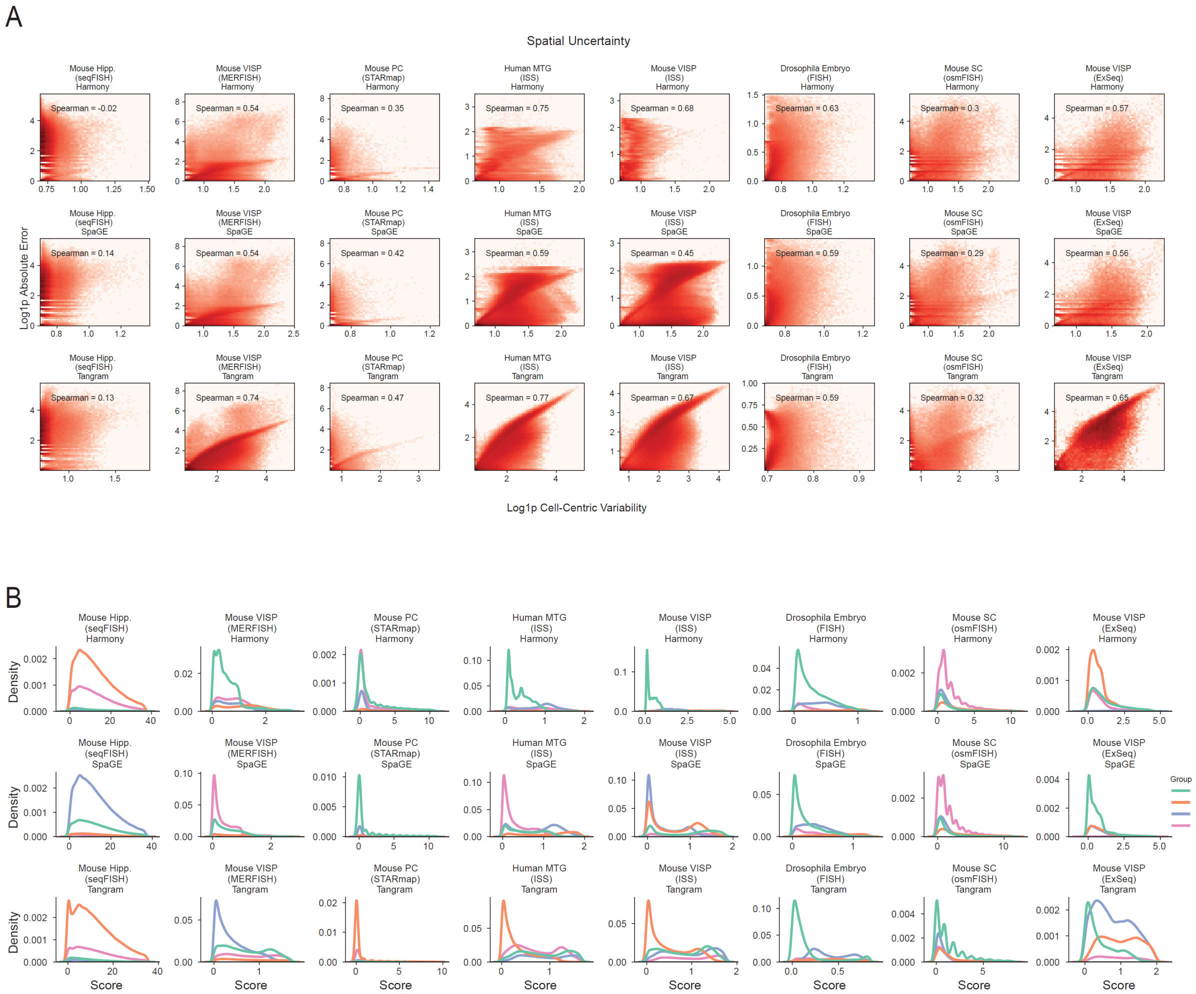

Figure S2: Individual cell-centric variability and calibration score distributions for all datasets and prediction methods. (A) Correlation of cell-centric variability and absolute prediction error shown individually for each dataset and prediction method combination computed over 10-fold cross-validation. Log density with added pseudocount (Log1p) is shown by color. (D) Distribution of TISSUE calibration scores shown individually for each dataset and prediction method combination ($(k_g, k_c) = (4, 1)$). Details on each dataset and prediction method can be found in Methods.

bioRxiv preprint doi: https://doi.org/10.1101/2023.04.25.538326; this version posted April 29, 2023. The copyright holder for this preprint (which was not certified by peer review) is the author/funder, who has granted bioRxiv a license to display the preprint in perpetuity. It is made available under aCC-BY 4.0 International license.

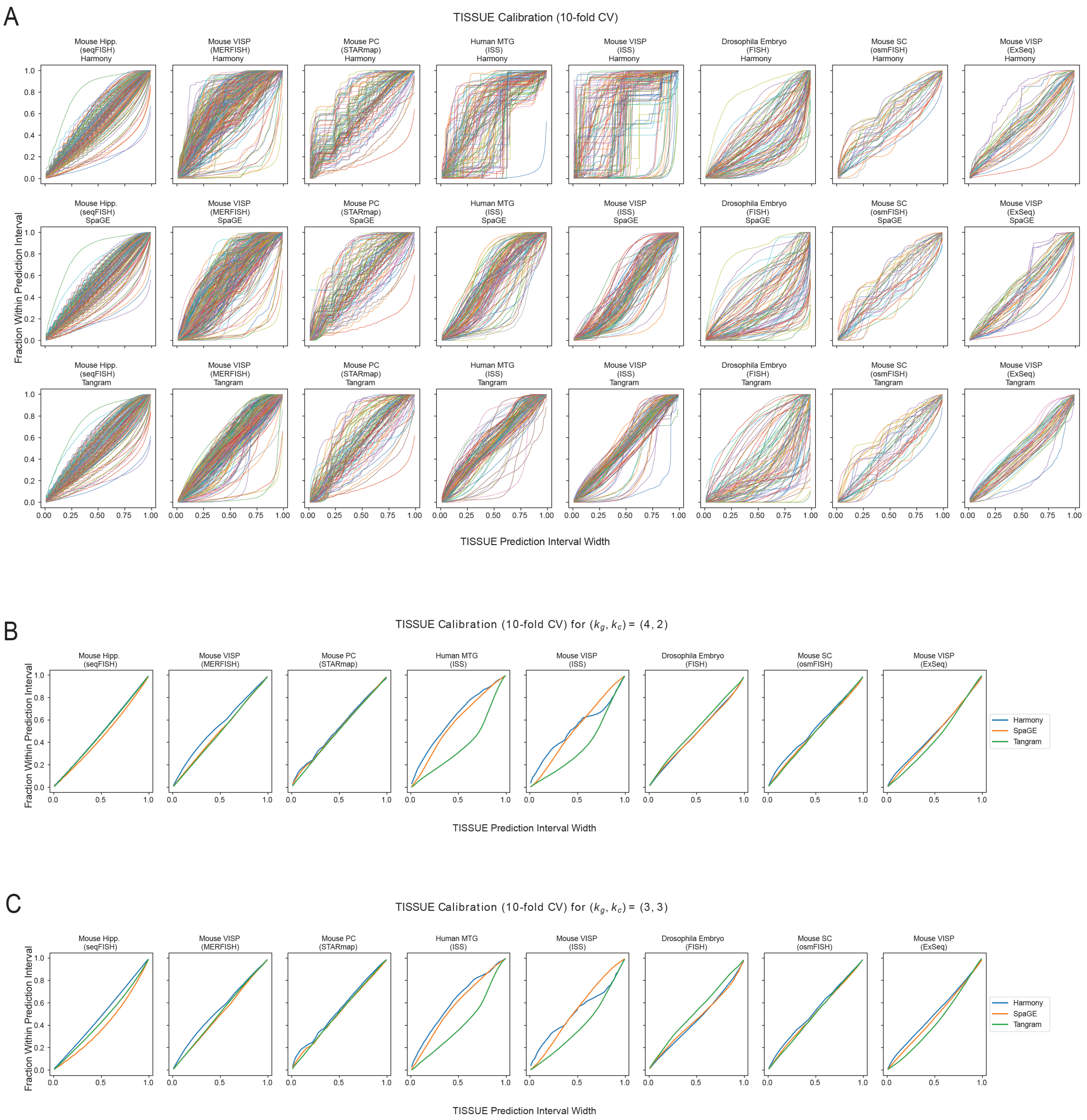

Figure S3: Additional prediction intervals for spatial gene expression. (A) Gene-level calibration curves for TISSUE prediction intervals showing empirical coverage as a function of the specified confidence level across 10-fold cross-validation. Each line corresponds to an independent gene in the spatial transcriptomics dataset. (B-C) Calibration curves for TISSUE prediction intervals showing empirical coverage as a function of the specified confidence level across 10-fold cross-validation for (B) $(k_g, k_c) = (4, 2)$ settings for stratified grouping, and (C) $(k_g, k_c) = (3, 3)$ settings for stratified grouping.

bioRxiv preprint doi: https://doi.org/10.1101/2023.04.25.538326; this version posted April 29, 2023. The copyright holder for this preprint (which was not certified by peer review) is the author/funder, who has granted bioRxiv a license to display the preprint in perpetuity. It is made available under aCC-BY 4.0 International license.

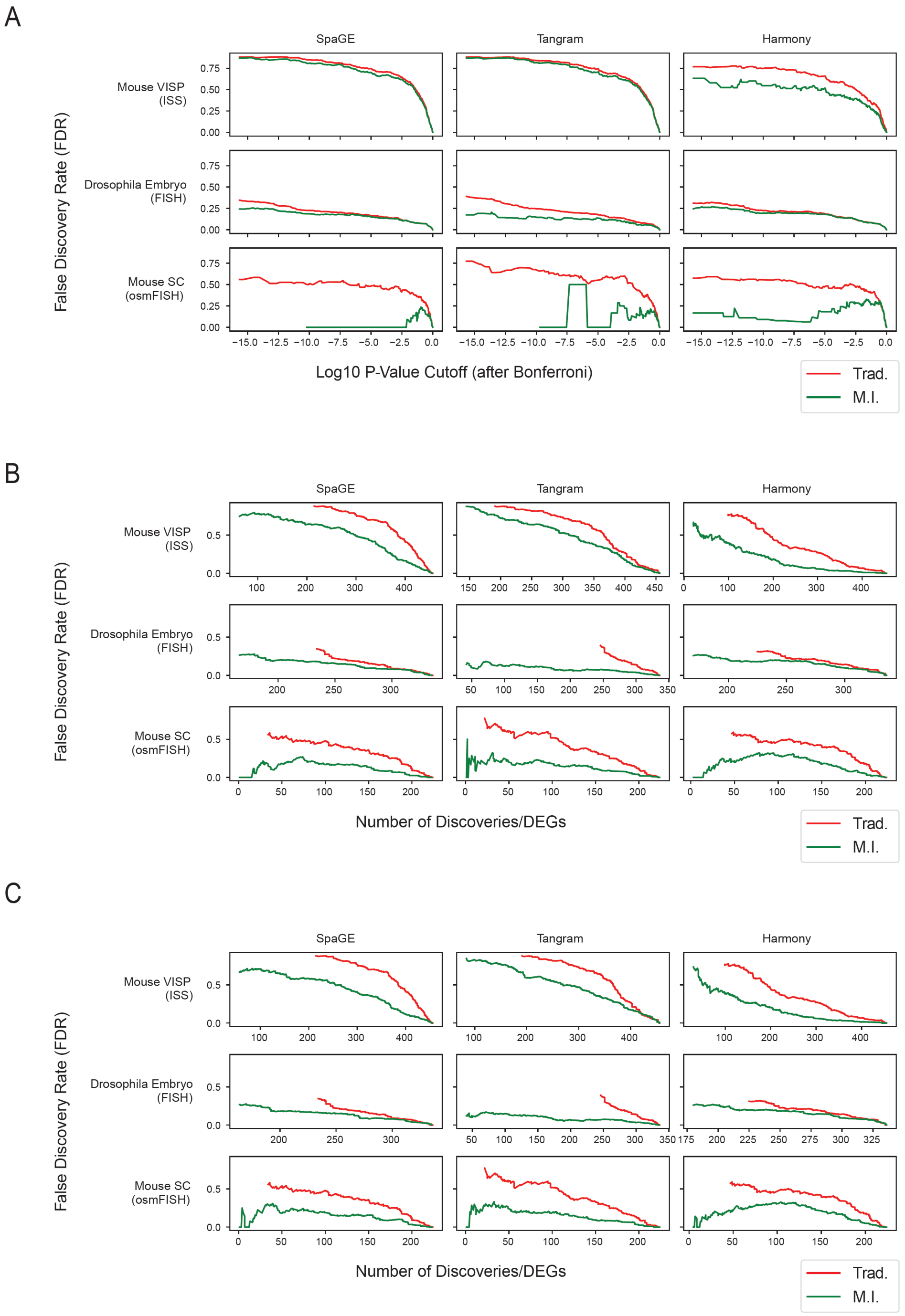

Figure S4: Additional differential gene expression analysis with TISSUE. (A) False discovery rate of differentially expressed genes between cell type or anatomic region labels (one versus all approach) as a function of the Bonferroni-corrected p-value threshold for significance and with $(k_g, k_c) = (4, 1)$ settings for stratified grouping. (B-C) False discovery rate of differentially expressed genes between cell type or anatomic region labels (one versus all approach) as a function of the number of discoveries and with (B) $(k_g, k_c) = (4, 2)$ settings for stratified grouping, and (C) $(k_g, k_c) = (3, 3)$ settings for stratified grouping.

bioRxiv preprint doi: https://doi.org/10.1101/2023.04.25.538326; this version posted April 29, 2023. The copyright holder for this preprint (which was not certified by peer review) is the author/funder, who has granted bioRxiv a license to display the preprint in perpetuity. It is made available under aCC-BY 4.0 International license.

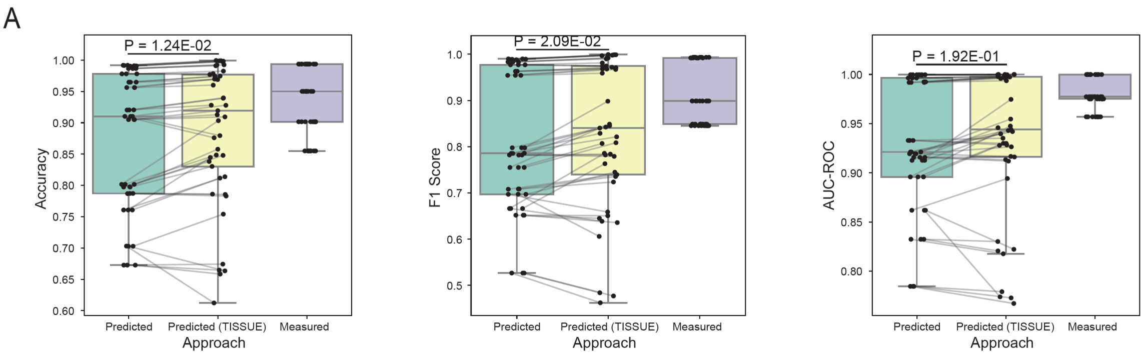

Figure S5: Aggregate performance of TISSUE-filtering for predictive modeling. (A) Accuracy, F1 score, and AUROC (area under the receiver-operator characteristic curve) metrics for logistic regression models trained on the predicted gene expression, TISSUE-filtered predicted gene expression, or measured gene expression for classification of the three most prominent class labels for three datasets. Shown are metrics for all three prediction methods, three different stratified grouping settings ($(k_g, k_c) = (4,1)$, $(k_g, k_c) = (4,2)$, $(k_g, k_c) = (3,3)$), and two sets of class labels (cell type and anatomic region). P-value was computed using a paired two-sample t-test.

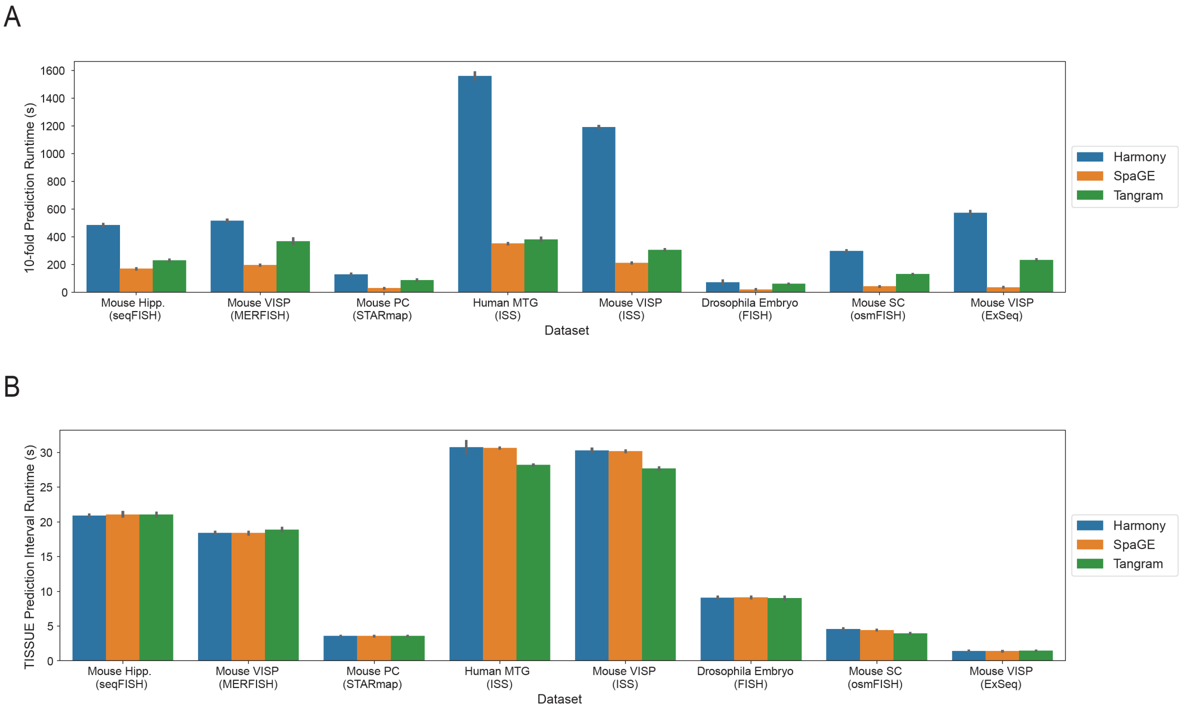

Figure S6: Computational runtime for TISSUE. (A) Bar plots of total runtimes for spatial gene expression prediction computations over 10 predictions to generate estimated predictions on all calibration genes. Error bars correspond to 95% confidence interval over an outer 10-fold cross-validation. (B) Bar plots of total runtimes for TISSUE prediction interval calculation including computation of cell-centric variability and calibration score sets. Error bars correspond to 95% confidence interval over an outer 10-fold cross-validation.
